# OpenCASA: A new open-source and scalable tool for sperm quality analysis

**DOI:** 10.1371/journal.pcbi.1006691

**Published:** 2019-01-18

**Authors:** Carlos Alquézar-Baeta, Silvia Gimeno-Martos, Sara Miguel-Jiménez, Pilar Santolaria, Jesús Yániz, Inmaculada Palacín, Adriana Casao, José Álvaro Cebrián-Pérez, Teresa Muiño-Blanco, Rosaura Pérez-Pé

**Affiliations:** 1 Department of Biochemistry and Molecular and Cellular Biology-Institute of Research in Environmental Sciences of Aragon (IUCA), Veterinary Faculty, University of Zaragoza, Zaragoza, Spain; 2 TECNOGAM Research Group, Institute of Research in Environmental Sciences of Aragon (IUCA), Higher Polytechnic School of Huesca, University of Zaragoza, Huesca, Spain; Worcester Polytechnic Institute, UNITED STATES

## Abstract

In the field of assisted reproductive techniques (ART), computer-assisted sperm analysis (CASA) systems have proved their utility and potential for assessing sperm quality, improving the prediction of the fertility potential of a seminal dose. Although most laboratories and scientific centers use commercial systems, in the recent years certain free and open-source alternatives have emerged that can reduce the costs that research groups have to face. However, these open-source alternatives cannot analyze sperm kinetic responses to different stimuli, such as chemotaxis, thermotaxis or rheotaxis. In addition, the programs released to date have not usually been designed to encourage the scalability and the continuity of software development. We have developed an open-source CASA software, called OpenCASA, which allows users to study three classical sperm quality parameters: motility, morphometry and membrane integrity (viability) and offers the possibility of analyzing the guided movement response of spermatozoa to different stimuli (useful for chemotaxis, thermotaxis or rheotaxis studies) or different motile cells such as bacteria, using a single software. This software has been released in a Version Control System at Github. This platform will allow researchers not only to download the software but also to be involved in and contribute to further developments. Additionally, a Google group has been created to allow the research community to interact and discuss OpenCASA. For validation of the OpenCASA software, we analysed different simulated sperm populations (for chemotaxis module) and evaluated 36 ejaculates obtained from 12 fertile rams using other sperm analysis systems (for motility, membrane integrity and morphology modules). The results were compared with those obtained by Open-CASA using the Pearson’s correlation and Bland-Altman tests, obtaining a high level of correlation in all parameters and a good agreement between the different used methods and the OpenCASA. With this work, we propose an open-source project oriented to the development of a new software application for sperm quality analysis. This proposed software will use a minimally centralized infrastructure to allow the continued development of its modules by the research community.

## Author summary

In the field of the biology of reproduction, it is very important to develop new methods that allow us to predict the breeding capacity of males of certain species. In recent years, the use of Computer Assisted Sperm Analysis systems (that allow the user to analyze certain parameters of sperm that have been shown to be related to fertility), has been fundamental in advancing this knowledge. In addition, new parameters are being investigated that are believed to help characterize seminal quality. However, most of the software used to analyze these parameters is commercial and private, and the user is not allowed to add new features. Although there were already open source alternatives to analyze certain parameters of seminal quality, in this work we have integrated several tools into one, with an easy-to-use interface for the user, which we hope will help the scientific community to develop new functionalities and methods. The tool is free and open-source.

This is a *PLOS Computational Biology* Software paper.

## Introduction

In the field of assisted reproductive techniques (ART), computer-assisted sperm analysis (CASA) systems have proved their utility and potential for analyzing sperm quality [1,2]. These systems have been used to determine the relationship between sperm motility [3], morphometry [4–6] or membrane integrity [7,8] and fertility rates. Besides good motility, proper morphology and membrane integrity, the sperm capacity to respond to the guidance mechanism toward the egg is an essential characteristic. In recent years it has become clear that mammalian spermatozoa must be guided to reach the oocyte, and three different mechanisms have been proposed to date, at least in human: thermotaxis [9], rheotaxis [10] and chemotaxis [11–13], each of which is a response to a specific stimulus: temperature gradient, fluid flow and concentration gradient, respectively. Currently it cannot be ruled out that the movement guided to the oocyte could be due to a combination of several of these mechanisms [14]. Recent studies provide experimental support for the importance of guidance in the fertilization process [15]. Thus, the study of the sperm responsiveness to these guidance mechanisms could be a good indicator of seminal quality and could help predict the fertility of a given seminal sample.

Although most researchers use commercial systems, certain free and open-source CASA alternatives have been developed during the last years. Thus, open-source software has been used for morphometry [5], motility [16–18] and membrane integrity [19] analysis. However, to the best of our knowledge, no open-source software for the analysis of sperm responsiveness to guidance mechanism has been released. These initiatives undoubtedly represent an advance within the open science framework [20–22], where the availability of both the software and the source code of the analysis programs is considered crucial to guarantee the reliability and reproducibility of a scientific study. However, despite their usefulness, these programs are still way behind the commercial CASA systems in terms of ease of use and standardization, and they have not usually been designed to encourage the scalability and the continuity of the software development. Hence, the source code is usually written in one single file and published by references to static web pages or by links to a file hosting service, like *Dropbox*. In this scenario, users can download the software but they cannot update or improve these programs for the benefit of other users. In the worst cases, the link is broken shortly after publication.

Thus, due to the lack of an open-source alternative for the analysis of sperm responsiveness to guidance mechanism, and in order to integrate several sperm quality parameters in the same tool and make the code reusable and scalable, we set out to develop a new open-source, sperm analysis software. We have tried to follow the good practices for computational science proposed by other authors [23,24], and thus the source code has been structured in different packages depending on its functionality. The software is released in a version control system that acts as a minimal centralized infrastructure. This platform will allow researchers not only to download the software but also to be involved in and contribute to further developments.

Thus, the aim of this study was to develop a free software that offers the possibility of analyzing several parameters related to seminal quality. This software, that we have named OpenCASA, includes four modules: motility, membrane integrity, morphology and a module specific for the study of the sperm guidance. To the latter, we have called it “chemotaxis module”, but it could be used to study the guided movement response of spermatozoa to any other stimulus (including thermotaxis and rheotaxis), or even for other cells with movement capacity, such as bacteria. Moreover, the software includes an additional module to simulate the chemoattracted sperm populations, or any other cell population being attracted by other stimuli. This simulation module can be useful for the validation of different methods related to the chemotaxis module, and it can also help beginners to understand how the chemotaxis module works, creating and analyzing populations with different levels of attraction. Once developed, the different modules were validated, either by simulation or by comparison with other analysis systems. The results indicated a good agreement between the different used methods and the OpenCASA. This agreement, together with the advantage of being open-source and incorporating novel analysis possibilities, such as the sperm movement response to stimuli, makes this new software a powerful tool for laboratories dedicated to semen analysis.

## Design and implementation

The OpenCASA software has been implemented in Java language. The starting point was the source code previously developed by Wilson-Leedy & Ingermann [16], which uses internally ImageJ libraries for image processing and analysis [25,26]. The software architecture was designed to facilitate the subsequent development of new features, so the code was separated in different packages depending on its functionality (Table 1). This categorization helps to reutilize the previously implemented code, e.g., functions related to the identification of cells in an image, or to extract kinetic parameters from a set of trajectories, and it makes the source code files shorter and less complex.

**Table 1:** Description of the software packages included in OpenCASA.

*Package*	Description
*Analysis*	This package includes code directly related to the implemented analysis.
*Data*	This package includes the implementation of new data structures.
*GUI*	This package includes the code related to the graphical user interface.
*Functions*	This package includes generic functions that could be used by every module, i.e. statistical functions, video processing or drawing functions.
*Third Party*	This package has been created to include and locate third party code used in the project.

**Fig 1:**
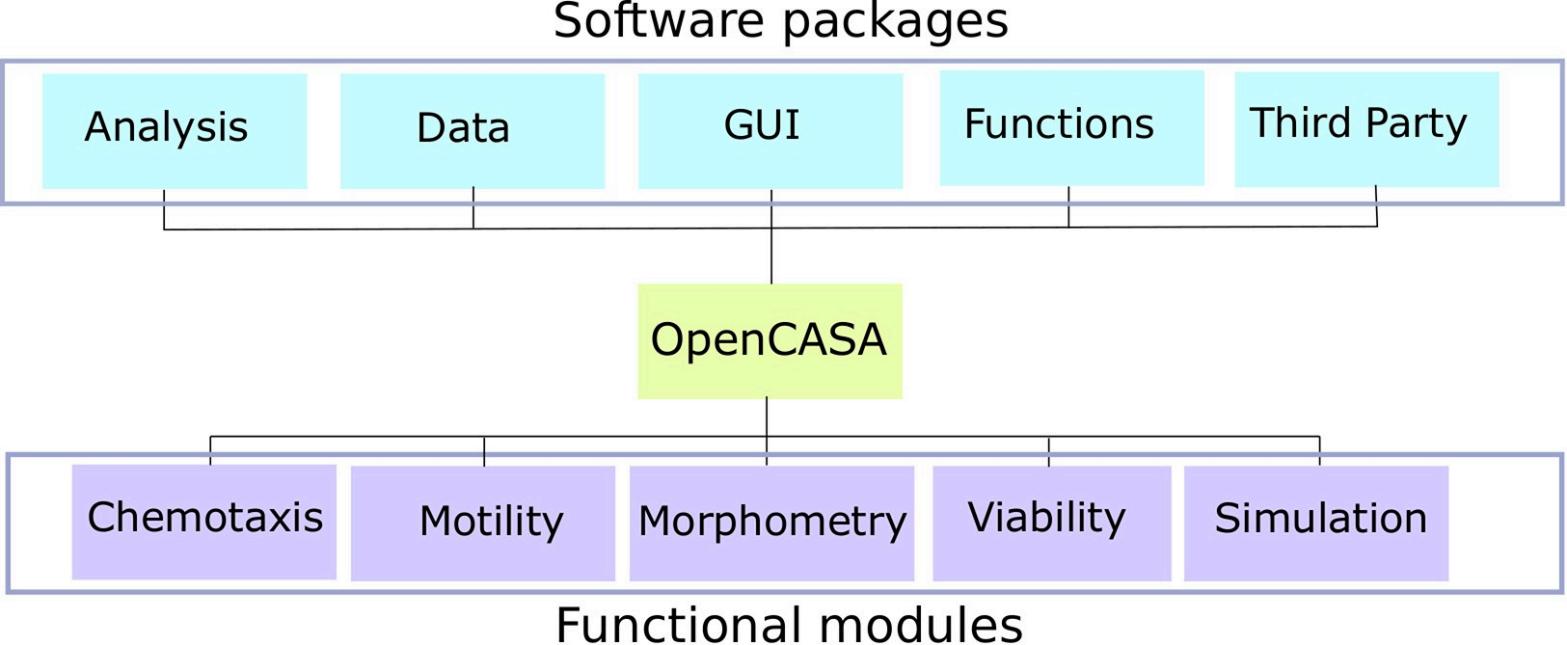
OpenCASA overview diagram. The software architecture was designed to facilitate the subsequent development of new features, so the code was separated in different packages depending on its functionality. Using these packages, the program allows user to carry out four different sperm analyses through the corresponding modules: Chemotaxis, Motility, Morphometry and Viability. In addition, a fifth module was implemented to generate simulations of chemotactically attracted sperm populations.

The program was initially designed to include four functional modules (Fig 1). Furthermore, during the software development an additional module to simulate chemoattracted sperm populations was created in order to validate the chemotaxis analysis.

All information related to the software design is comprised in the Software Design Document (SDD) included as supplemental file of this manuscript.

### Motility module

The plugin developed by Wilson-Leedy & Ingermann [16] that we used as the source code allowed us to calculate several kinematic parameters. However, we refactored the code in order to make it simpler and more readable, including a new set of kinematic functions in the “Functions” software package (Fig 1). The new module allows us to calculate total and progressive motilities, curvilinear velocity (VCL), linear velocity (VSL), average path velocity (VAP), linearity coefficient (LIN), wobble coefficient (WOB), straightness coefficient (STR), mean and maximum amplitude of lateral head displacement (ALH), frequency of head displacement (BCF), dance (DNC) and mean angular displacement (MAD). The equations and definitions are summarized in Table 2. Moreover, we have included the parameter “fractal dimension” (FD), which serves the best for characterizing hyperactivated motility and structure of trajectories in general [27,28] which is not included in other CASA systems.

**Table 2:** Mathematical definition and meaning of the kinematic parameters implemented in the OpenCASA software.

Parameter	Mathematical definition	Meaning (from Mortimer [29])
Straight-line velocity (VSL)	$D(p_{1},p_{N})*\frac{FrameRate}{N-1}*\mu$	VSL is determined by finding the straight-line distance between the first and last points of the trajectory and correcting for time. This value then gives the net space gain within the observation period
Curvilinear velocity (VCL)	$\left(\sum_{t=1}^{N-1}D(p_{t},p_{t+1})\right)*\frac{FrameRate}{N-1}*\mu$	VCL is the distance travelled by the spermatozoon along its curvilinear path/s and is calculated by finding the sum of the distances along the trajectory then correcting for time. It refers to the total distance that the sperm head covers in the observation period
Average-path velocity (VAP)	$\left(\sum_{t=1}^{N-w}D(q_{t},q_{t+1})\right)*\frac{FrameRate}{N-w}*\mu$	VAP is the distance the spermatozoon has traveled in the average direction of movement in the observation period. It is calculated by finding the length of the average path and correcting for time.
Linearity (LIN)	$\frac{VSL}{VCL}*100$	LIN is a comparison of the straight-line and curvilinear paths. It is an expression of the relationship between the two-dimensional projection of the three-dimensional path taken by the spermatozoon (i.e. curvilinear path) and its net space gain
Wobble (WOB)	$\frac{VAP}{VCL}*100$	WOB is the expression of the relationship between the average and curvilinear paths
Straightness (STR)	$\frac{VSL}{VAP}*100$	STR is a comparison of the straight-line and average paths and gives an indication of the relationship between the net space gain and the general trajectory of the spermatozoon
Amplitude of lateral head displacement *ALH*_*mean*_	Let the segment *S*_*t*_(formed by two consecutive points between *p*_*t*_ *and p*_*t*+*w*−1_) be the nearest segment to the segment S(*q*_*t*_,*q*_*t*+1_) relative to its middle point ${qm}_{t}=\frac{q_{t}+q_{t+1}}{2}$. ${ALH}_{mean}=2*\frac{\sum_{t=2}^{N-w}F(D({qm}_{t},S_{t}))}{Count}*\mu$ Where function F() and parameter Count are defined as: Global var Count = 0; // it counts the total number of elements that the function F() identify as local maxima. Function F(): If *D*(*qm*_*t*_,*S*_*t*_)>*D*(*qm*_*t*−1_,*S*_*t*−1_) and *D*(*qm*_*t*_,*S*_*t*_)>*D*(*qm*_*t*+1_,*S*_*t*+1_) then Count = Count+1 return *D*(*qm*_*t*_,*S*_*t*_) else return 0	The amplitude of lateral head displacement (ALH) is used as an approximation of the flagellar beat envelope. It is not a true amplitude, in that it does not measure the perpendicular distance between the peak of a wave and the point of inflection of the curve, but rather gives the distance between the ‘peak’ and ‘trough’ of the centroid’s path. ALHmean is the mean of all of the ALH values along the trajectory.
*ALH*_*max*_	Using the above definition: ${ALH}_{max}=2*{Max}_{t=2}^{N-w}[F(D({qm}_{t},S_{t}))]*\mu$	ALHmax is the maximum ALH found along the trajectory.
Beat-cross frequency (BCF)	Let *S*(*p*_1_,*p*_2_) be a function that returns the segment made by the points *p*_1_ and *p*_2_. *s*_1_ and *s*_2_ being two different segments, let us define *δ* as $\delta (s_{1},s_{2})=\left\{ \begin{array}{c} 1\;if\;s_{1}\cap s_{2}\\ 0\;otherwise \end{array}\right.$ Let $P={\left\{S(p_{t},p_{t+1})\right\}}_{t=1}^{N-1}$ be the set of all consecutive segments that form a trajectory, and $Q={\left\{S(q_{z},q_{z+1})\right\}}_{z=1}^{N-w}$ the set of all consecutive segments that form the corresponding average path for the trajectory {*p*_*t*_}. $BCF=\sum_{t,z}\delta (s_{p},s_{q})*\frac{FrameRate}{N-w}$, with *s*_*p*_∈*P*,*s*_*q*_∈*Q*	BCF is the number of times the sperm head crosses the direction of movement, and this is related to the development of another flagellar wave
DANCE (DNC)	VCL*ALH_*mean*_	DNC is a measure of the pattern of sperm motion VCL×ALH
Mean angular displacement (MAD)	$\frac{\sum_{t=1}^{N-\Delta }\hat{v_{t}}}{N-\Delta }$	MAD is a measure of the trajectory curvature, defined as ‘the time average of absolute values of the instantaneous turning angle of the head along its curvilinear trajectory’
Progressive Motility (PM)	Yes if STR > % and VAP> value, both defined by the user. Otherwise the trajectory is not considered progressive.	PM refers to sperm that are swimming in a mostly straight line
Motility trajectories	A trajectory is considered motile if the VCL parameter is greater than a value defined by the user (minimum vcl), and also the starting point of the trajectory is different than the last point.	This parameter refers to spermatozoa that are considered motile.
Fractal dimension (FD)	$FD=\frac{log(n)}{\left[\log\left(n\right)+log(\frac{d}{L})\right]}$ Where n is the number of track intervals (number of track points -1), d is the planar extent of the curve (maximum distance between the starting point and any other point of the track) and L is the length of the curvilinear path.	The fractal dimension is an expression of the degree to which a line fills a plane. It may be considered that the fractal dimension of a curve indicates its regularity. A curve with a low fractal dimension would be regular and predictable. Similarly, a curve with a high fractal dimension would have irregularly spaced changes in direction, apparently at random.

Let ${\left\{p_{t}\right\}}_{t=1}^{N}$ be a trajectory of length N defined as a sequence of N points; D(p,q) the Euclidean distance between the points p and q; and μ the scale factor (microns/pixel). Also let ${\left\{q_{t}\right\}}_{t=1}^{N-w+1}$ be the average trajectory of ${\left\{p_{t}\right\}}_{t=1}^{N}$ calculated applying a simple moving average with a rectangular window of size w ($q_{t}=\frac{1}{w}*\sum_{t}^{t+w-1}p_{t}$). Finally, let ${\hat{v}}_{t}$ be the angle of the vector specified by the points ⟨*p*_*t*_,*p*_*t*+Δ_⟩, with Δ a positive integer lower than N.

In addition, this module allows to analyze restricted time interval of each video, selecting in the motility settings at which second the user wants to start and/or finish the analysis. This option could be applied to a single video, a complete folder or multiple folders.

### Chemotaxis module

**Fig 2:**
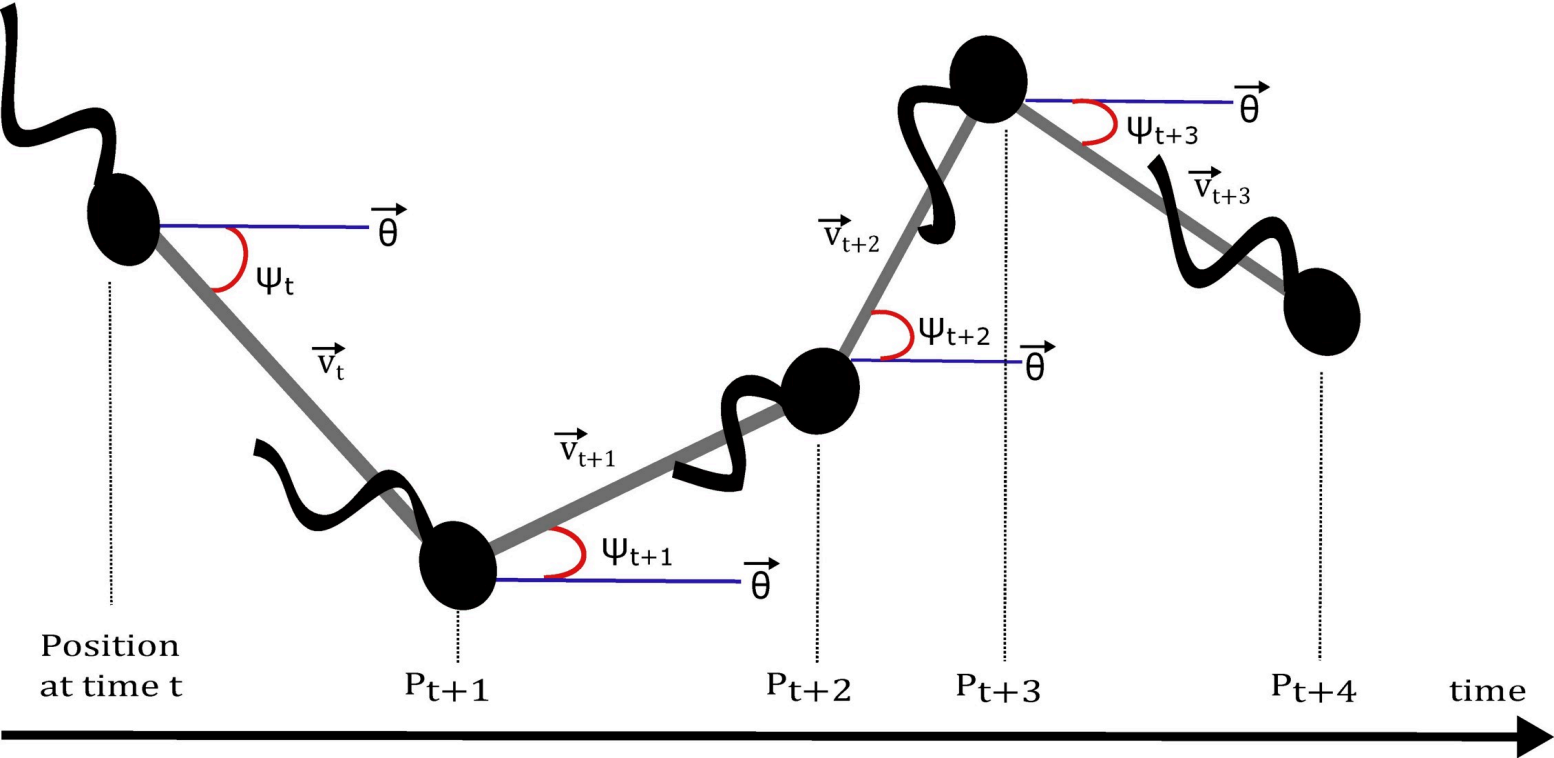
Definition of the instantaneous directionality angle. *ψ* is the angle between the vector of the cell frame-to-frame displacement (${\vec{v}}_{t}=\langle P_{t},P_{t+1}\rangle$) and the gradient direction $\vec{\theta }$. In the example above, the gradient has been set to $\vec{\theta }=0{}^{\circ}$.

Various chemotaxis chambers or devices are used in the study of chemotaxis where spermatozoa are loaded in one reservoir and a putative chemoattractant agent is loaded in another connected reservoir, so that spermatozoa are able to move in the chemoattractant direction or not. In order to evaluate the sperm chemotactic responsiveness, the program developed in this study analyses the bias in the directionality of the sperm movement on the basis of the distribution of the instantaneous directionality angles (*ψ*). *ψ* is the angle between the vector of the cell frame-to-frame displacement and the gradient direction $\vec{\theta }$ (Fig 2). When *ψ* is in the interval [−*γ*,+*γ*], it is assumed that chemoattraction exists, being *γ* a user-defined parameter that represents the amplitude of the chemoattractant gradient.

**Fig 3:**
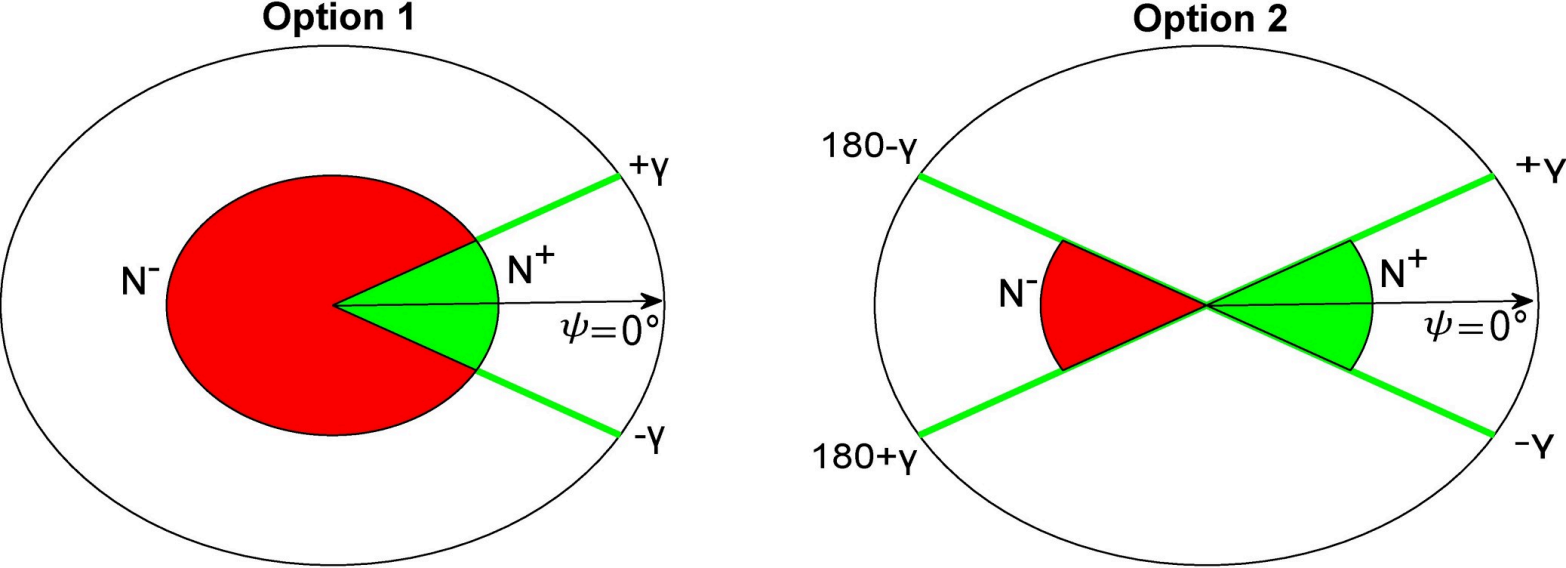
Definition of two different options in order to analyse the chemotaxis phenomena. It is important to count the number of instantaneous displacements ${\vec{v}}_{t}$ pointing in the chemotaxis gradient direction $\vec{\theta }$ and the number of those displacements not pointing to the gradient. $\psi =\langle \vec{v},\vec{\theta }\rangle$ being the angle between the instantaneous displacement of a cell and the gradient direction; *N*^+^ is defined as *count*{*ψ*∈[−*γ*,+*γ*]}, where *γ* is a parameter defined by the user and represents the amplitude of the chemoattractant concentration gradient. The developed software allows users to choose two options to define which displacements not pointing to the gradient are taken into account. In option 1, N^−^ is defined as *count*{*ψ*∉[−*γ*,+*γ*]}, whereas in option 2 only displacements in the opposite direction of the gradient are considered (*N*^−^ = *count*{*ψ*∈[180°−*γ*,180°+*γ*]}). The images above show graphically which angles are taken into account for the sum *N*^+^(green color) and *N*^−^(red color), depending on the option specified by the user.

Due to the existence of different chemotaxis chambers, the software allows us to define which is the gradient direction and which displacements not pointing in this direction are considered (Fig 3). The chemotaxis analysis implemented here is based on the total number of displacements both pointing to the chemotaxis gradient (*N*^+^) and not pointing to that gradient (*N*^−^).

**Fig 4:**
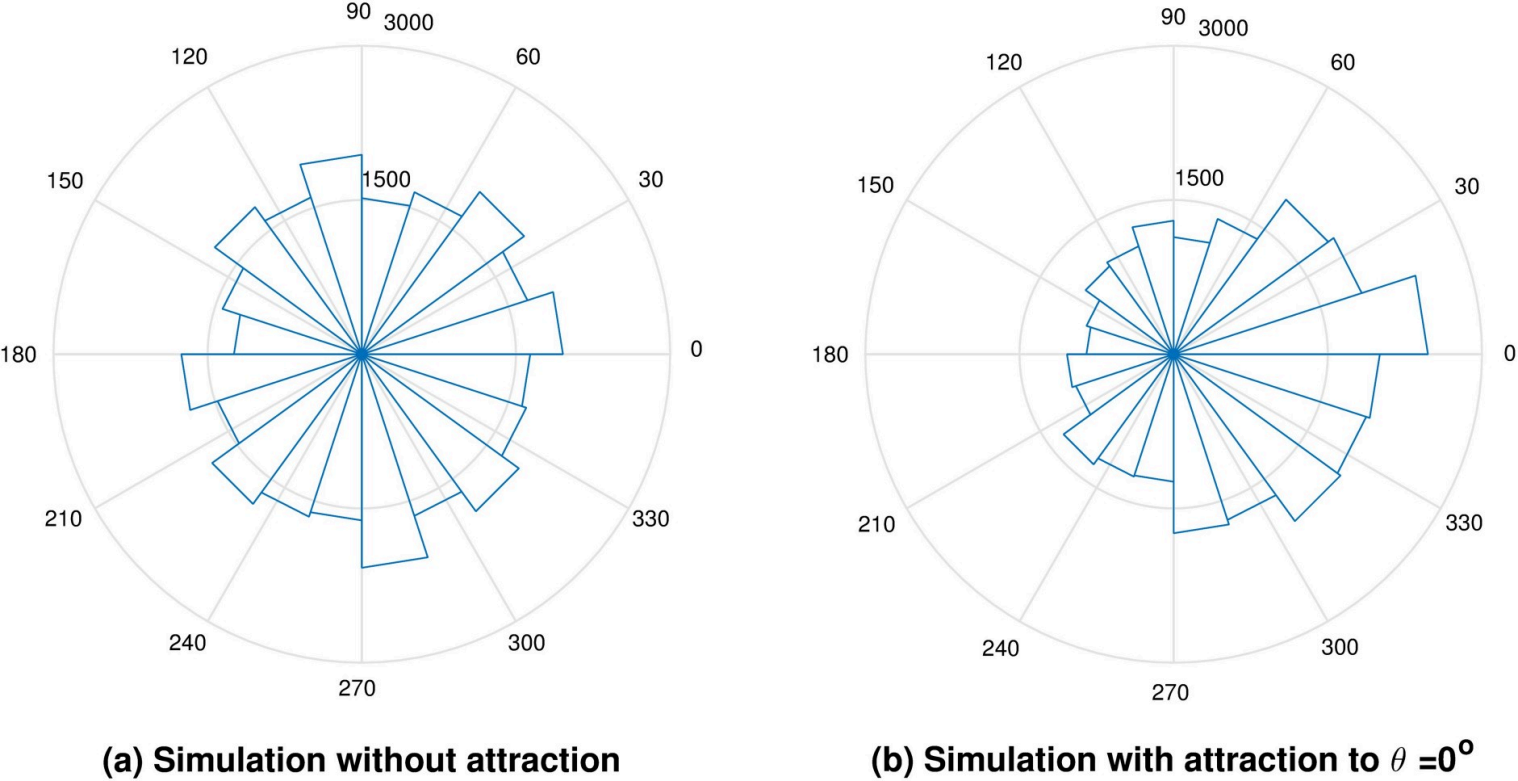
Two examples of the distribution of the instantaneous directionality angles *ψ*. **(a) Simulated sperm population without chemotaxis. (b) Simulated sperm population chemoattracted to *θ* = 0° (right).** The *ch-index* provides information about the percentage of the angles *ψ* that point in the gradient direction with respect to the total number of angles taken into account (the total number of angles will depend on the option specified by the user). The parameters used to generate the simulation on the right were β = 1 and Responsiveness = 50%.

Once all sperm tracks have been identified in a recorded video, the chemotaxis module provides information about the percentage of instantaneous displacements pointing in the chemoattractant gradient direction with respect to those not pointing in the same direction ($Ch_{Index}=\frac{N^{+}}{N^{+}+N^{-}}*100$, Fig 4).

Furthermore, it allows us to detect non-uniform distributions of sperm swimming directionality angles (using *bootstrapping analysis*).

For the *bootstrapping analysis*, we adapted the bootstrapping method described by Armon *et al*. [12] for detecting non-uniform distributions of sperm swimming directionality angles. Firstly, the odds value is defined as the ratio between the number of displacements *N*^+^ pointing in the chemoattractant gradient direction and the number of displacements *N*^−^ not pointing towards the gradient $\left(Odds\;Value=\frac{N^{+}}{N^{-}}\right)$. Then, we defined the O.R. ratio as the fraction of the odds value calculated from the sperm trajectories recorded in a chemotaxis test, divided by the odds value from the sperm trajectories coming from a control condition. This ratio measures the strength of the chemotactic phenomena in the gradient direction. Values close to one would mean that the attraction in the chemotactic test is similar to that obtained in the control, while values greater than one would indicate a chemoattraction toward the chemotactic gradient.

O.R.=Odds_ValuetestOdds_Valuecontrol=N+N−Nc+Nc− (1)

**Fig 5:**
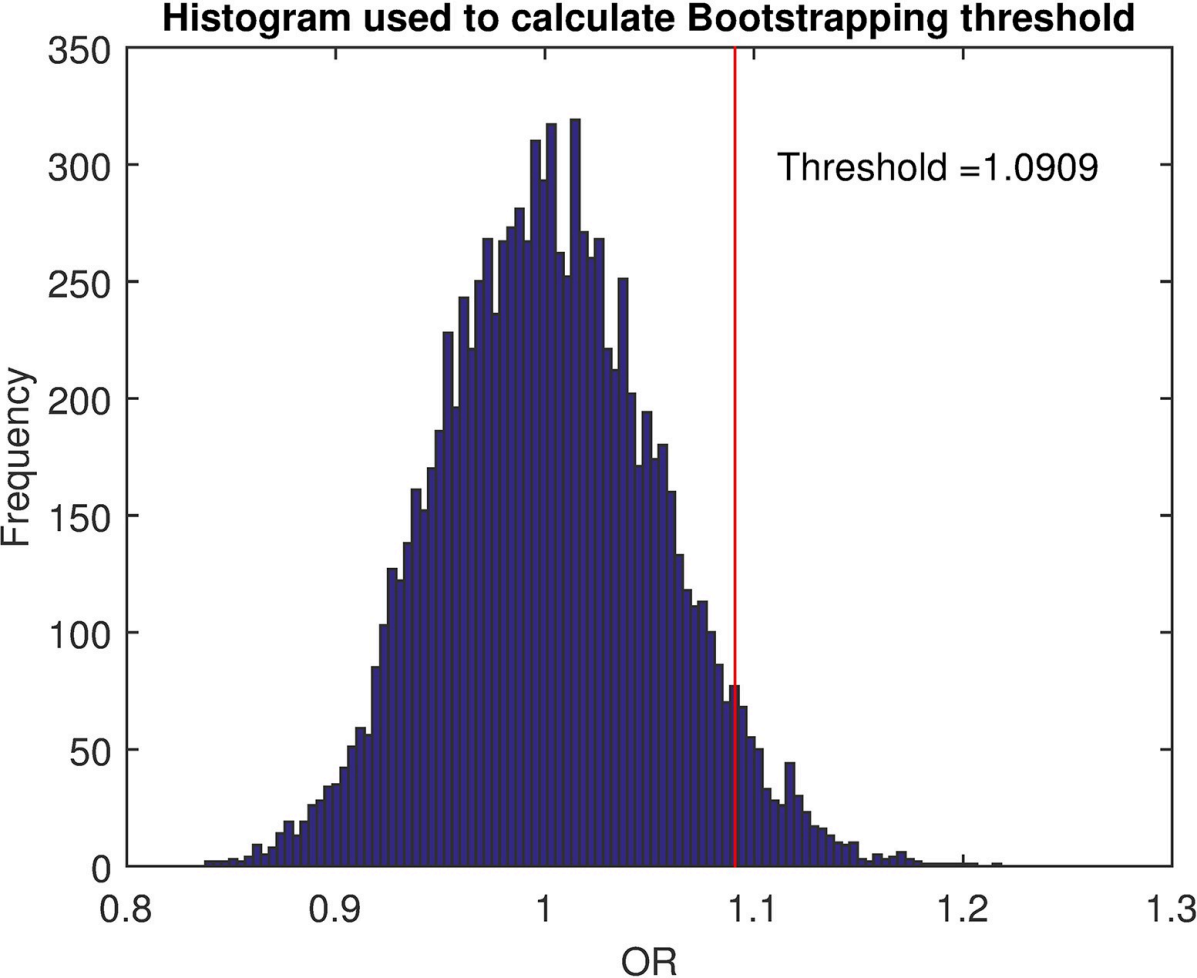
Determination of the O.R. threshold used to discriminate between chemotaxis and no chemotaxis in the bootstrapping method. The histogram comes from 10000 O.R. ratios, each one calculated by the odds value of two disjointed subsets of trajectories randomly sampled over all detected trajectories in 100 control simulations. Each simulation consisted of a 500 frames length video (800x800 pixels each frame) containing 100 virtual cells randomly located at the beginning of the simulation. Each cell was defined as an ellipse (10x8 pixels size) and behaved following a persistent random walk equation with parameters *D*_*rot*_ = 0.1, *v*_0_ = 3,*β* = 0, *Reponsiveness* = 0 *and ψ*_0_ = 0°.

Theoretically, the O.R. ratio should be 1 when two controls are compared, although some bias in this ratio can be found due to the finite sampling and the noise produced by the intersection of the sperm trajectories in the track-recognition step [12]. In order to characterize this variability, the method takes into account all trajectories corresponding to neutral recordings, and calculates several times the O.R. ratio for two disjointed subsets of trajectories randomly sampled (with replacement after each iteration). Once the distribution is calculated, the threshold to determine when an O.R. ratio indicates chemoattraction needs to be set (Fig 5). In OpenCASA, this threshold has been set as the percentile *P*_95_. Under experimental conditions, when a control situation is compared with another using a putative chemoattractant agent, a chemotaxis phenomenon is assumed to exist if the O.R. ratio between them is greater than the threshold.

### Simulation module

This module simulates sperm populations that follow the persistent random walk model specified by Armon *et al*. [12]. The mathematical equations that govern this behavior are:

$$\frac{d\psi }{dt}=-\left(\frac{\beta }{\tau }\right)\sin(\psi )+\xi \sqrt{2D_{rot}}$$ (2)

$$\frac{dx}{dt}=v_{0}\cos\left(\psi \right)$$ (3)

$$\frac{dy}{dt}=v_{0}\sin\left(\psi \right)$$ (4)

As the authors describe, this persistent random walk model is characterized by a swimming angle direction *ψ*(*t*). The instantaneous changes in that angle depend on a rotational diffusion coefficient (*D*_*rot*_), and the term $\left(\frac{\beta }{\tau }\right)\sin\left(\psi \right)$ provides the simulated cells with the ability to align their path to a uniform external field parallel to the x-axis at a rate $\frac{\beta }{\tau }$ (the bigger the parameter *β*, the stronger the attraction effect). Also, the positive or negative sign of the *β* parameter will determine the right/left direction of the cells, whereas *β* = 0 means no attraction. Finally, *v*_0_ defines the (constant) swimming speed and *ξ*(*t*) denotes Gaussian white noise with variance 1.

It is worth mentioning that, in mammalian semen only a small fraction of the cells, the capacitated spermatozoa, is chemotactically attracted by the oocyte [13,14]. Thus, we introduced a new parameter in our simulations, called “responsiveness”, which represents the percentage of capacitated cells in our simulated sperm population.

### Membrane integrity (viability) module

**Fig 6:**
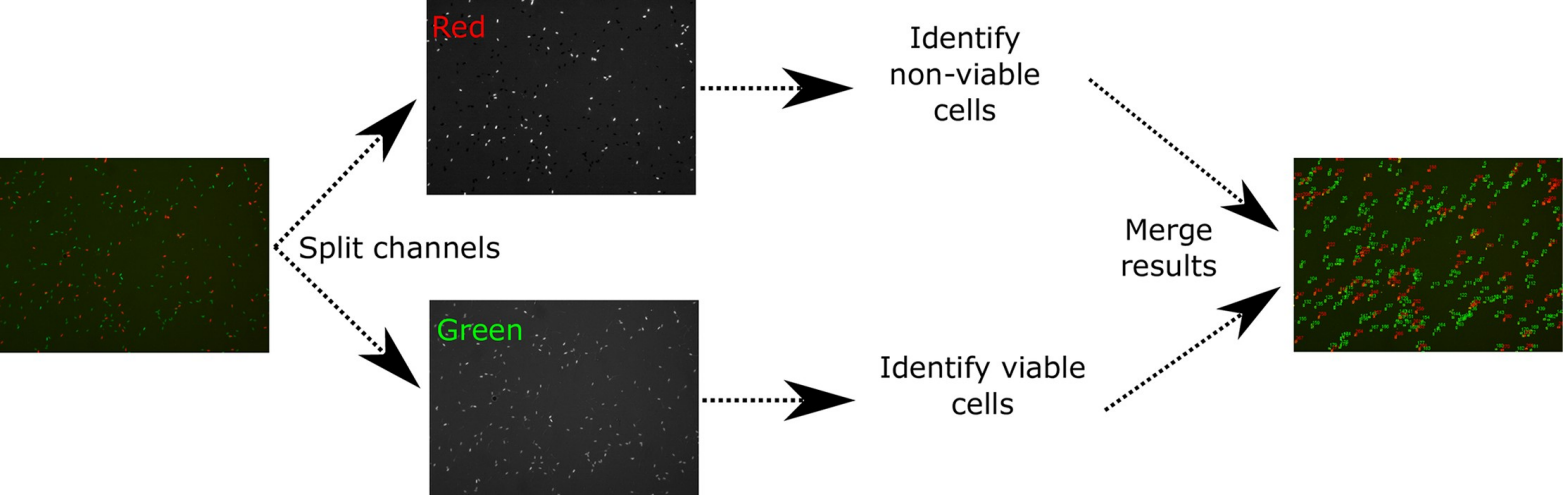
Membrane integrity module workflow. The module receives an RGB image as input, splits the image into red and green channels, identifies viable and non-viable cells depending on the channel and finally merges all the results showing all the detected cells in the same image. The module identifies the viable cells in green and the non-viable cells in red.

The membrane integrity module works with images and is based on the identification of the color of a set of previously stained cells. The program counts the number of green and red cells contained in a given image, splitting it between the red and green RGB channels (Fig 6). Over each of these channels, the program identifies and labels each cell as viable or non-viable depending on the source channel (viable for green cells and non-viable for red cells).

### Morphometry module

This module implements a semi-automated process to measure the morphometry of sperm heads captured in an image. For each cell, the program determines the area, perimeter, length, width, ellipticity, roughness, elongation and regularity. The equations or definitions are summarized in Table 3.

**Table 3:** Definition of the morphometric parameters implemented in the OpenCASA software.

Parameter	Definition
Mean gray value	Average gray value of all pixels contained in the cell area (value between 0 and 255).
Area	Area of the cell (μm^2^).
Perimeter	Perimeter of the cell (μm).
Length	Length of the cell following the principal axis. Equivalent to Feret value (μm).
Width	Width of the cell following the secondary axis. Equivalent to Min Feret (μm).
Ellipticity	$\frac{Length}{Width}$
Roughness	$\frac{4*\pi *Area}{{Perimeter}^{2}}$
Elongation	$\frac{Length-Width}{Length+Width}$
Regularity	$\frac{Length*Width*\pi }{4*Area}$

### Technical issues and limitations of the OpenCASA software

This program has been developed and tested on Windows 7 (64-bit) using ImageJ v1.49q and Java JRE 1.8.0_101 (64-bit). There are no specific requirements to use this plugin, but a special attention of RAM memory is suggested when video analysis is carried on. At least 5GB of heap memory size is recommended, but it depends on the size of the files. One good estimation could be to use a heap memory size of 2.5 times the size of the heaviest file that is going to be analysed. The plugin has not been tested on Linux or MAC platforms.

For video analysis, OpenCASA works with AVI format. There are no restrictions for resolution and frame rate, but it is necessary to take into account that the number of trajectories, their length and the number of points per trajectory will depend on the sampling frequency. For validation, videos were recorded at 60 fps during 1 second with a resolution of 768x576 pixels.

For image analysis, OpenCASA works well with JPEG format. As in video analysis, there are no restrictions about the resolution, but the user will obtain a better performance with higher pixel density. Images used for validation had a resolution of 1936 x 1288 pixels.

Regarding the general parameters, the maximum and minimum cell size will depend on cell’s type and species. The minimum VCL will be used to determine if a cell is motile or not. Cells with trajectories with a VCL lower than this value will be labelled as non-motile cells. Also, the program will remove automatically those tracks with a length lower than the “Minimum Track Length” parameter. This parameter cannot be higher than the total number of frames in the video. The “Maximum displacements between frames” is a parameter used to determine how much microns a cell can move from one frame to the next. It defines the search area of the same cell on the next frame. Cells inside this area will be evaluated as candidates and cells outside this area will be ignored. The parameter “Window size” is the size of the rectangle window used in the moving average method applied to calculate the average path. This parameter has to be in a range between 2 and the total number of frames minus 1.

It is worth noting that the value of these parameters will have to be adapted depending on the frame rate (frames/second: fps) used to video recording. For example, in case of using a higher frame rate than 60 fps (the frame rate used in the validation of this software), the value of the parameter “Maximum displacements between frames” should be decreased because a cell will take a shorter displacement between a frame and the following one. Conversely, with lower frame rates, this parameter will need to be increased.

In Motility analysis, if the size of the files is too big, it could take a lot of time to draw the trajectories in the output video shown at the end of the execution. The VCL values lower and upper threshold are used to define levels of motility in terms of their curvilinear velocity and to colour the trajectories in the output video (white for slow trajectories, yellow for moderate trajectories, and red for faster trajectories). It is important to consider that these thresholds may need to be changed depending on the frame rate used to record the videos, due to the VCL increase with higher frame rates.

In Chemotaxis analysis, the bootstrapping resamples determine the number of mixings and recalculations of the OR ratio in order to build the distribution of the index and to choose and appropriate threshold to discriminate later between positive and non-attracted displacements. It is recommended not to modify this parameter unless the user has statistical notions about the bootstrapping analysis. When the videos have been recorded with a high frame rate, the software usually do not detect cell’s motion between frames. In order to detect a displacement in some direction, it is a good option to add some delay between both frames used to calculate the direction. The parameter “Angle Delta” has the number of frames that are skipped in the middle. The minimum value for this parameter is 1 and the maximum has to be lower than the total number of frames minus 1.

### Experimental design and statistical analyses for validation of OpenCASA

For validation of the OpenCASA chemotaxis module, we simulated a high number of sperm populations using the persistent random walk model described above. For validation of the rest of OpenCASA modules (motility, membrane integrity and morphology), we used 36 ejaculates obtained from 12 fertile rams during May 2017. Ejaculates were collected individually using sterilized artificial vaginas and glass tubes (IMV L'Aigle. France). Spermatozoa were diluted in INRA96 (IMV L'Aigle. France) extender to a final concentration of 30 x 10^6^ cells/mL, packaged in glass tubes, and stored at 15 ºC until sperm quality assessment. All experimental procedures were performed under Project License PI19/17 approved by the Ethics Committee for Animal Experiments of the University of Zaragoza.

Samples were analyzed by other image analysis systems (ISAS, Version 1.1, PROISER, Valencia, Spain, for motility and Image J for morphometry) or by flow cytometry (for viability), and the results were compared with those obtained by OpenCASA using the Pearson’s correlation test (GraphPad Prism 5, GraphPad Software, Inc., La Jolla, CA, USA). However, given that the correlation studies the relationship between one variable and another, not the differences, a Bland-Altman test (GraphPad Prism 5, GraphPad Software, Inc., La Jolla, CA, USA) was carried out to study the agreement between the different measurement systems. For the Bland-Altman test, the difference vs. average was plotted for those sperm parameters expressed as percentage (i.e. viability, motility, progressive motility…), whereas the percentage difference vs. average was used for continuous variables (i.e. morphometry parameters, VCL, VSL…).

## Results

### Validation of chemotaxis module

**Fig 7:**
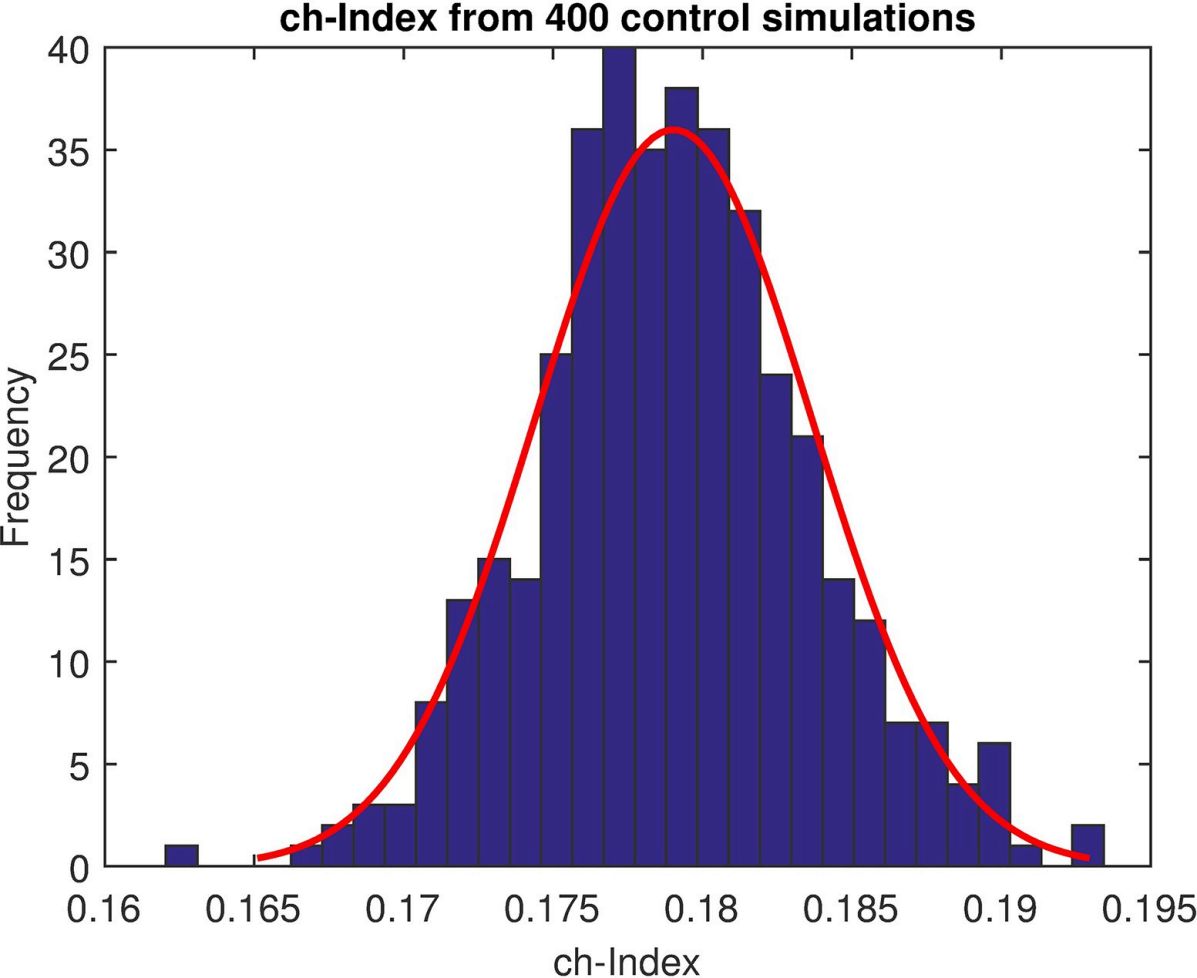
Verification of the *ch-index* in a non-chemotaxis condition. The *ch-index* provides information about the percentage of the angles *ψ* that point in the gradient direction with respect to the total of angles taken into account. In the case of a non- chemotaxis condition, a uniform distribution of the instantaneous directionality angles is expected, so defining *γ* = 30° and considering the angles *ψ* in the range [−30°,+30°] as chemotactical responses to the gradient, theoretically the percentage of those angles with respect to the total number of angles would be $\frac{60{}^{\circ}}{360{}^{\circ}}*100\approx 16.67\%$. Analysing the histogram, as expected, the *ch-index* was centred close to the theoretical value (17.90±0.46%) with some variation due to the sampling and the noise of the system (e.g. intersection of trajectories).

In all the analyses carried out, the gradient direction $\vec{\theta }$ was assumed to be at 0º (positive x-axis). First, we simulated a control sperm population without any chemoattraction (*β* = 0). Considering the total number of displacements within the range [−30°,+30°], we calculated the percentage of angles obtained in that range over the total number of angles (option 1, Fig 3). In a neutral (non-biased) situation, with a completely random distribution of the instantaneous displacements, the expected number of angles within the set range should be $\frac{60{}^{\circ}}{360{}^{\circ}}*100\approx 16.67\%$. The *ch-index* was calculated from 400 runs (each simulation consisted of 100 simulated cells moving on a video of 500 frames length). As expected, the *ch-index* was close to the theoretical value (17.90±0.46%) with some variation due to the sampling and the noise of the system (Fig 7).

In order to confirm the correct functioning of the module, we also performed a *bootstrapping* analysis. First, we analysed the distribution of the O.R. ratios calculated in the process to set the O.R. threshold. Simulating 100 different sperm populations without any bias in the directionality, we calculated the O.R. ratios of 10000 resamplings (Fig 5). As expected, the distribution of these ratios was centred at 1 (1.0024±0.0523). Then, considering *P*_95_ as the O.R. threshold, one should expect a theoretical 5% possible false positives in any analysis. Keeping this in mind, we paired up each previous simulation with a new one (also with no bias in the directionality), and we calculated the corresponding O.R. ratio in order to determine how many of these simulations were labeled as positive in chemotaxis. As expected, only 5 false positives (5%) were found in the 100 O.R. ratios obtained. Tests with bias in the directionality (*β*>0) resulted in a higher number of positives, i.e., more O.R. ratios upper O.R. threshold.

### Validation of motility module

Sperm motility was first evaluated by a commercial computer-assisted sperm analyzer (ISAS, Version 1.1, PROISER, Valencia, Spain). Two consecutive drops and at least 500 sperm cells were analyzed using a pre-warmed Makler chamber (Sefi-Medical Instruments, Haifa, Israel) and under an Olympus BX40 microscope with a 10X magnification objective. The settings were as follows: frames per second: 60; number of frames: 60; VCL threshold: 10 μm/s. The image sequences obtained were also evaluated with the motility module of the OpenCASA program. The motility results obtained with both the commercial analyzer and our CASA program were compared using Pearson’s correlation test. All motility values obtained with our OpenCASA software correlated highly with those obtained with the commercial program and, in general, showed a good agreement between both measurement systems on the basis of the Bland-Altman test (Table 4).

**Table 4:** Comparison between the motility parameter values given by ISAS or by OpenCASA using a Pearson’s correlation test and a Bland-Altman test.

	*Pearson´s Correlation test*		*Bland-Altman*
*Motility Parameters*	*r*	*p-Value*	*Bias (%)*
*Motile trajectories*	0.9756	< 0.0001	-1.84
*VSL*	0.9677	< 0.0001	11.37
*VCL*	0.9402	< 0.0001	-1.0
*VAP*	0.9671	< 0.0001	6.38
*LIN*	0.9466	< 0.0001	5.82
*WOB*	0.9659	< 0.0001	4.50
*STR*	0.8767	< 0.0001	3.26
*ALH*	0.8794	< 0.0001	-12.69
*BCF*	0.8779	< 0.0001	20.75
*Progressive Motility*	0.8180	< 0.0001	3.50
*Motility*	0.9606	< 0.0001	-2.57

### Validation of viability module

Sperm membrane integrity was determined by means of acridine orange (AO) and propidium iodide (PI) [19] using the DUO-VITAL kit (Halotech, Madrid, Spain). Samples were placed in a Makler chamber (Sefi-Medical Instruments, Haifa, Israel). In order to validate the OpenCASA viability module, the images of at least 200 spermatozoa per sample were captured by fluorescence microscopy. Digital images of the fluorescence-labelled sperm were obtained using an epifluorescence microscope (Leica DM4500B, Wetzlar, Germany) under a 10X magnification objective, equipped with the appropriate filter sets. A JenOptik ProgRes CF CCD (JenOptik AG, Jena, Germany) coupled with JENOPTIK PROGRES CAPTURE PRO image acquisition software was used. The percentage of viable and non-viable spermatozoa was evaluated by Open-CASA software, and the same samples were assessed by flow cytometry. The flow cytometry analyses were performed on a Beckman Coulter FC 500 (Beckman Coulter Inc., Brea, CA, USA) with CXP software. A minimum of 20000 events were counted in all the experiments. A flow rate stabilized at 200–300 cells per second was used. Monitored parameters were FS log, SS log, FL1 (525 nm), and FL4 (675 nm). The sperm population was gated for further analysis on the basis of its specific forward (FS) and side scatter (SS) properties, so other non-sperm events were excluded. The flow cytometry and OpenCASA viability results were compared using Pearson’s correlation test, and a highly significant correlation (P<0.0001) was obtained for viable and non-viable spermatozoa (Table 5). The Bland-Altman test showed a very good agreement between both measurement systems (Table 5).

**Table 5:** Comparison between the membrane integrity results obtained by flow cytometry or by OpenCASA using a Pearson’s correlation test and a Bland-Altman test.

	*Pearson´s Correlation test*		*Bland-Altman*
Viability parameters	*r*	*p-Value*	*Bias (%)*
*Viable*	0.7901	< 0.0001	-6.99
*Non-Viable*	0.7987	< 0.0001	9.16

### Validation of morphometry module

Semen smears were prepared for sperm morphometry assessment as previously described in Yániz et al. [30]. Briefly, semen smears were allowed to air dry for a minimum of 2 h, fixed with 2% glutaraldehyde in PBS for a 3-min exposure, washed thoroughly in distilled water and labelled with Hoechst. Samples were stained by placing 20 μL of a Hoechst 33342 suspension (20 μg/ml in a TRIS-based solution) between the slide and a coverslip, which was then incubated for dark at RT. The coverslip was then removed, and the slide was washed thoroughly with distilled water and allowed to dry.

In order to validate the OpenCASA morphometry module, the sperm morphometry was first determined using the CASMA-F method [30] and a Leica DM4500B microscope with a 63X plan-apochromatic objective. At least 200 sperm cells were recorded per sample and analyzed by Image J, and the same images were afterwards processed with the OpenCASA software. The results corresponding to area, perimeter, intensity (mean gray value), length (Feret) and width (minFeret) were compared using Pearson’s correlation test showing a highly significant correlation (P<0.0001) in all sperm morphology parameters (Table 6). The Bland-Altman test showed a very good agreement between both measurement systems (Table 6).

**Table 6:** Comparison between the values of the morphometry parameters given by CASMA-F or by OpenCASA using a Pearson’s correlation test and a Bland-Altman test.

	*Pearson´s Correlation test*		*Bland-Altman test*
Morphometry parameters	*r*	*p-Value*	*Bias (%)*
*Area*	0.8226	< 0.0001	0.39
*Perimeter*	0.8982	< 0.0001	-0.21
*Intensity*	0.9348	< 0.0001	2.88
*Feret (Length)*	0.9368	< 0.0001	0.11
*MinFeret(Width)*	0.8387	< 0.0001	0.19

## Availability and future directions

The software has been released at Github (https://github.com/calquezar/OpenCASA). This platform will allow researchers not only to download the software, but also to be involved in and contribute to further developments. Additionally, a test data has been uploaded to figshare (https://doi.org/10.6084/m9.figshare.7247039.v1), and a Google group has been created to allow the community to interact and discuss OpenCASA further, and access the group via the forum (https://groups.google.com/d/forum/opencasa_mailinglist) or via the mailing list (opencasa_mailinglist@googlegroups.com). Both user’s manual and test instructions have been uploaded to the Github repository.

This work presents the first version of an open-source computer assisted sperm analysis (CASA) system named OpenCASA. Commercial CASA systems are costly, present low flexibility in order to modify some features, and most of them deal with motility uniquely [1,2,18,31,32] To date, different plugins for free image analysis programs, such as the image-J, allowed to perform, separately, the analysis of one parameter, such as motility or morphometry [17,30]. OpenCASA allows, using a single software structured in four different modules, to analyze motility, viability, morphology and the sperm response of guidance mechanism.

This software was developed using ram sperm as a model. Nevertheless, the flexibility of the system allows, by modifying the parameters, to be used for other species and cell types. However, it is important to know that different settings may highly affect the results obtained by CASA, especially when it is done in absolutely automatic way. In order to determine the settings for the analysis of a new cell types, we recommend validating the obtained results by comparison with another analysis system, as we have done in this work. It is worth pointing out that this could be a starting point for future collaborations between groups that work with different species which could communicate using the channels established here (email list or forum). A good contribution of these collaborations to the community would be to stablish by consensus the standards for different species.

For the validation of the motility, morphometry and viability modules, we have compared our OpenCASA with specific analysis systems, commercial or not (ISAS, CASMA-F or flow cytometry). As a future work it would be interesting to make a comparison between the results provided by Open CASA and other different analysis systems to see the degree of adjustment between them [1,2,18,31,32].

In the motility module, apart from the classical kinetics parameters included in other commercial or free CASA systems, we have included the parameter named “fractal dimension” (FD), which serves the best for characterizing hyperactivated motility and structure of trajectories in general [27,28].

Regarding to the module for the study of sperm response of guidance mechanism, we have named “chemotaxis module” on the basis of the use we usually make. But actually, the module analyzes changes in the directionality of the trajectories, which can be in response to a gradient of a chemical substance, temperature, or a fluid flow. Therefore, it could be used to study the guided movement response of spermatozoa to any other stimulus, including thermotaxis and rheotaxis. Therefore, the module could also have been called "Sperm Directionality Analysis Module", or even “Directionality Analysis Module” as it could be applied not only to sperm but to any cell with movement capacity in response to a stimulus, such as bacteria (by previous setting of the particle size parameters).

However, recent advances in the study of rheotaxis include the use of microfluidic devices where both the sperm and the fluid itself have been constantly moving [33]. In this case, the OpenCASA software may not be adequate in its current configuration and may require modifications, as Elsayed *et al*. carried out from a plugin for Image-J [33].

Thus, due to the lack of an easy-to-use and open-source tool for the study of the sperm guided movement, and the importance of this mechanism in the fertilization process [14,15,34] we trust in the potentiality of this module. Moreover, the software includes an additional module to simulate the chemoattracted sperm populations, or any other cell population being attracted by other stimuli. This simulation module could be useful for the validation of the chemotaxis module in any other condition.

Unlike other plugins developed for the study of certain cell quality parameters, the development of the OpenCASA software does not end here but remains open for the incorporation of new modules and new functionalities. For this purpose, we would like to appeal to the scientific community to collaborate and use the communication networks mentioned above.

In conclusion, we presented OpenCASA, a new free software for the analysis of several quality parameters of the seminal samples. OpenCASA allows, using a single software structured in four different modules, to analyze motility, viability, morphology and the sperm response of guidance mechanism. Modules were validated, either by simulation with an additional module included in the software, or by comparison with other analysis systems. The results indicated a good agreement between the different used methods and the OpenCASA. The software is released in a version control system that acts as a minimal centralized infrastructure. This platform will allow researchers not only to download the software but also to be involved in and contribute to further developments. All these advantages make OpenCASA a powerful tool for laboratories dedicated to semen analysis.

## Supporting information

S1 Fig Bland-Altman test for motility parameters. Results obtained for motility in the validation test.
(TIF)

S2 Fig Bland-Altman test for morphometry parameters. Results obtained for morphometry in the validation test.
(TIF)

S3 Fig Bland-Altman test for viability parameters. Results obtained for viability in the validation test.
(TIF)

S1 File Software Design Document. This document contains the specification of some algorithms, data structures and activity diagrams with the purpose to provide an overall view of the OpenCASA software design and its implementation.
(PDF)

## References

[1] Mortimer ST. CASA—Practical Aspects. J Androl. 2000 7 8;21(4):515–24. 10901437

[2] Amann RP, Waberski D. Computer-assisted sperm analysis (CASA): Capabilities and potential developments. Theriogenology. 2014 1 1;81(1):5–17.e3. 10.1016/j.theriogenology.2013.09.004 24274405

[3] Hirano Y, Shibahara H, Obara H, Suzuki T, Takamizawa S, Yamaguchi C, et al ANDROLOGY: Relationships Between Sperm Motility Characteristics Assessed by the Computer-Aided Sperm Analysis (CASA) and Fertilization Rates In Vitro. J Assist Reprod Genet. 2001 4 1;18(4):215–20. 10.1023/A:1009420432234 PMC3455361 11432113

[4] Ombelet W, Menkveld R, Kruger TF, Steeno O. Sperm morphology assessment: historical review in relation to fertility. Hum Reprod Update. 1995 1 1;1(6):543–57. 9079395 10.1093/humupd/1.6.543

[5] Butts IAE, Ward MAR, Litvak MK, Pitcher TE, Alavi SMH, Trippel EA, et al Automated sperm head morphology analyzer for open-source software. Theriogenology. 2011 12 1;76(9):1756–1761.e3. 10.1016/j.theriogenology.2011.06.019 21962916

[6] de Paz P, Mata-Campuzano M, Tizado EJ, Álvarez M, Álvarez-Rodríguez M, Herraez P, et al The relationship between ram sperm head morphometry and fertility depends on the procedures of acquisition and analysis used. Theriogenology. 2011 10 15;76(7):1313–25. 10.1016/j.theriogenology.2011.05.038 21798583

[7] Garner DL, Pinkel D, Johnson LA, Pace MM. Assessment of Spermatozoal Function Using Dual Fluorescent Staining and Flow Cytometric Analyses. Biol Reprod. 1986 2 1;34(1):127–38. 3955132 10.1095/biolreprod34.1.127

[8] Harrison R a. P, Vickers SE. Use of fluorescent probes to assess membrane integrity in mammalian spermatozoa. J Reprod Fertil. 1990 1 1;88(1):343–52. 1690300 10.1530/jrf.0.0880343

[9] Bahat A, Eisenbach M. Sperm thermotaxis. Mol Cell Endocrinol. 2006 6 27;252(1):115–9. 16672171 10.1016/j.mce.2006.03.027

[10] Miki K, Clapham DE. Rheotaxis guides mammalian sperm. Curr Biol CB. 2013 3 18;23(6):443–52. 10.1016/j.cub.2013.02.007 23453951 PMC3607503

[11] Armon L, Eisenbach M. Behavioral Mechanism during Human Sperm Chemotaxis: Involvement of Hyperactivation. PLOS ONE. 2011 12 7;6(12):e28359 10.1371/journal.pone.0028359 22163296 PMC3233563

[12] Armon L, Caplan SR, Eisenbach M, Friedrich BM. Testing Human Sperm Chemotaxis: How to Detect Biased Motion in Population Assays. PLoS ONE [Internet]. 2012 3 8 [cited 2017 Jan 16];7(3). Available from: http://www.ncbi.nlm.nih.gov/pmc/articles/PMC3297605/10.1371/journal.pone.0032909 PMC3297605 22412947

[13] Gakamsky A, Schechtman E, Caplan SR, Eisenbach M. Analysis of chemotaxis when the fraction of responsive cells is small—application to mammalian sperm guidance. Int J Dev Biol. 2004 9 1;52(5–6):481–7. 10.1387/ijdb.072520ag 18649261

[14] Eisenbach M, Giojalas LC. Sperm guidance in mammals—an unpaved road to the egg. Nat Rev Mol Cell Biol. 2006 4;7(4):276–85. 10.1038/nrm1893 16607290

[15] Pérez-Cerezales S, Laguna-Barraza R, Castro AC de Sánchez-Calabuig MJ, Cano-Oliva E, Castro-Pita FJ de, et al Sperm selection by thermotaxis improves ICSI outcome in mice. Sci Rep. 2018 2 13;8(1):2902 10.1038/s41598-018-21335-8 29440764 PMC5811574

[16] Wilson-Leedy JG, Ingermann RL. Development of a novel CASA system based on open source software for characterization of zebrafish sperm motility parameters. Theriogenology. 2007 2 1;67(3):661–72. 10.1016/j.theriogenology.2006.10.003 17137620

[17] Purchase CF, Earle PT. Modifications to the imagej computer assisted sperm analysis plugin greatly improve efficiency and fundamentally alter the scope of attainable data. J Appl Ichthyol. 2012 12 1;28(6):1013–6.

[18] Giaretta E, Munerato M, Yeste M, Galeati G, Spinaci M, Tamanini C, et al Implementing an open-access CASA software for the assessment of stallion sperm motility: Relationship with other sperm quality parameters. Anim Reprod Sci. 2017 1 1;176:11–9. 10.1016/j.anireprosci.2016.11.003 27887759

[19] Yániz J, Palacín I, Vicente-Fiel S, Gosalvez J, López-Fernández C, Santolaria P. Comparison of Membrane-Permeant Fluorescent Probes for Sperm Viability Assessment in the Ram. Reprod Domest Anim. 2013 8 1;48(4):598–603. 10.1111/rda.12132 23293961

[20] Dutton WH, Jeffreys PW. World Wide Research [Internet]. MIT Press 2010 [cited 2017 Feb 5]. Available from: https://mitpress.mit.edu/books/world-wide-research

[21] Woelfle M, Olliaro P, Todd MH. Open science is a research accelerator. Nat Chem. 2011 10;3(10):745–8. 10.1038/nchem.1149 21941234

[22] Munafò MR, Nosek BA, Bishop DVM, Button KS, Chambers CD, Sert NP du, et al A manifesto for reproducible science. Nat Hum Behav. 2017 1 10;1:0021. 10.1038/s41562-016-0021 PMC7610724 33954258

[23] Stodden V, Miguez S. Best Practices for Computational Science: Software Infrastructure and Environments for Reproducible and Extensible Research. J Open Res Softw [Internet]. 2014 7 9 [cited 2017 Dec 22];2(1). Available from: http://openresearchsoftware.metajnl.com/articles/10.5334/jors.ay/

[24] Wilson G, Aruliah DA, Brown CT, Hong NPC, Davis M, Guy RT, et al Best Practices for Scientific Computing. PLOS Biol. 2014 1 7;12(1):e1001745 10.1371/journal.pbio.1001745 24415924 PMC3886731

[25] Schindelin J, Rueden CT, Hiner MC, Eliceiri KW. The ImageJ ecosystem: An open platform for biomedical image analysis. Mol Reprod Dev. 2015 7 1;82(7–8):518–29. 10.1002/mrd.22489 26153368 PMC5428984

[26] Schneider CA, Rasband WS, Eliceiri KW. NIH Image to ImageJ: 25 years of image analysis. Nat Methods. 2012 7;9(7):671–5. 22930834 10.1038/nmeth.2089 PMC5554542

[27] Mortimer ST, Swan MA, Mortimer D. Fractal analysis of capacitating human spermatozoa. Hum Reprod Oxf Engl. 1996 5;11(5):1049–54. 10.1093/oxfordjournals.humrep.a019295 8671389

[28] Boryshpolets S, Pérez-Cerezales S, Eisenbach M. Behavioral mechanism of human sperm in thermotaxis: a role for hyperactivation. Hum Reprod. 2015 4;30(4):884–92. 10.1093/humrep/dev002 25609239

[29] Mortimer ST. A critical review of the physiological importance and analysis of sperm movement in mammals. Hum Reprod Update. 1997 10;3(5):403–39. 9528908 10.1093/humupd/3.5.403

[30] Yániz JL, Vicente-Fiel S, Capistrós S, Palacín I, Santolaria P. Automatic evaluation of ram sperm morphometry. Theriogenology. 2012 4 15;77(7):1343–50. 10.1016/j.theriogenology.2011.10.039 22225689

[31] Lu JC, Huang YF, Lü NQ. Computer-aided sperm analysis: past, present and future. Andrologia. 2014 5;46(4):329–38. 10.1111/and.12093 23550608

[32] Holt W, Watson P, Curry M, Holt C. Reproducibility of computer-aided semen analysis: comparison of five different systems used in a practical workshop**Data obtained at a British Andrology Society workshop on Computer-assisted Sperm Motility Analysis at the Royal Veterinary College, London, United Kingdom, September 24 to 25, 1992. Fertil Steril. 1994 12 1;62(6):1277–82. 7958000

[33] Elsayed M, El-Sherry TM, Abdelgawad M. Development of computer-assisted sperm analysis plugin for analyzing sperm motion in microfluidic environments using Image-J. Theriogenology. 2015 11 1;84(8):1367–77. 10.1016/j.theriogenology.2015.07.021 26318232

[34] Suarez SS, Pacey AA. Sperm transport in the female reproductive tract. Hum Reprod Update. 2006 2;12(1):23–37. 10.1093/humupd/dmi047 16272225

