# Supplementary figures and images for: OpenCASA: A new open-source and scalable tool for sperm quality analysis
Source: PLoS Comput Biol. 2019 Jan 18;15(1):e1006691. doi: 10.1371/journal.pcbi.1006691 (PMC6355034; doi:10.1371/journal.pcbi.1006691)

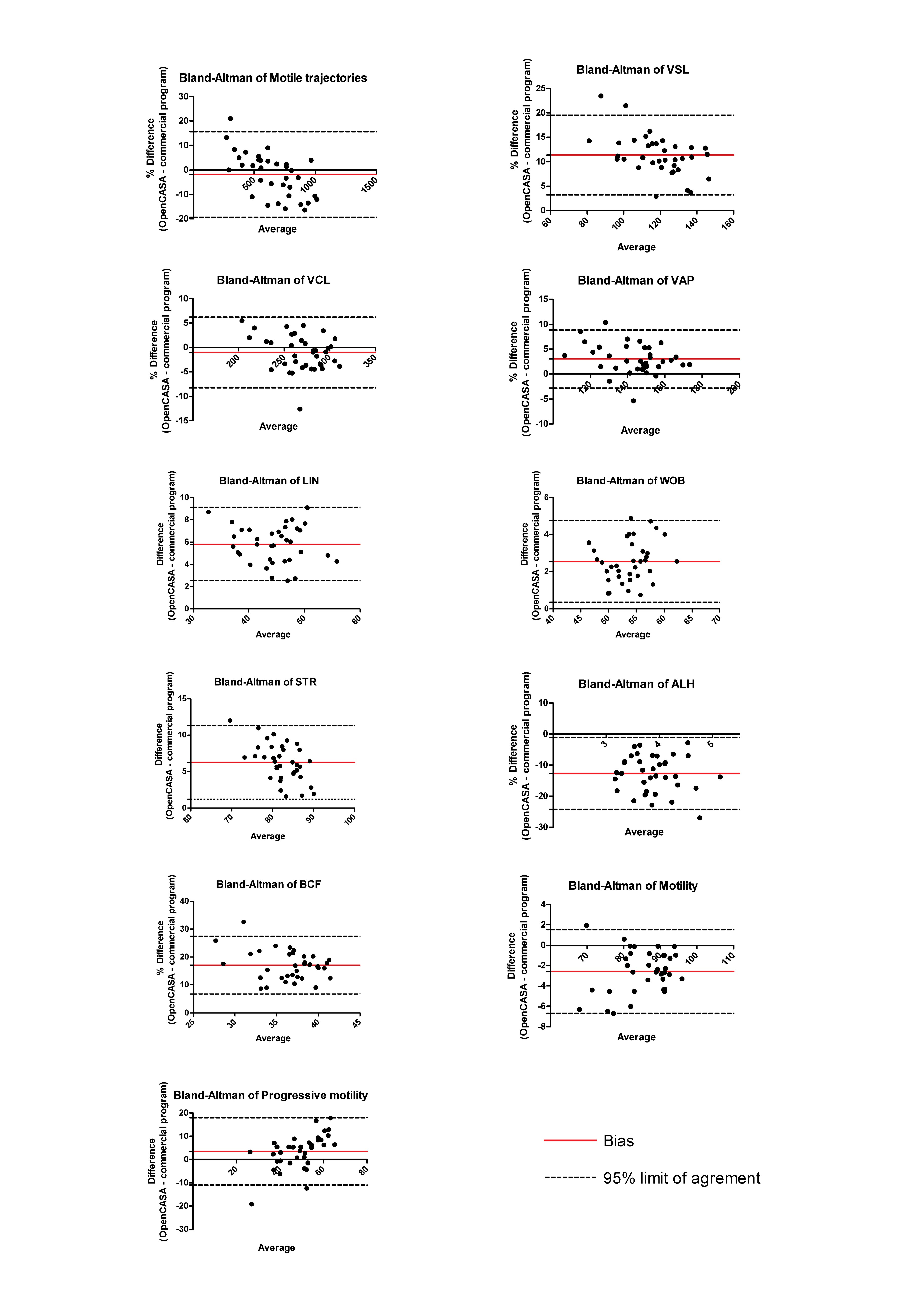

Supplement: S1 Fig — Results obtained for motility in the validation test. (TIF) [file pcbi.1006691.s001.tif]

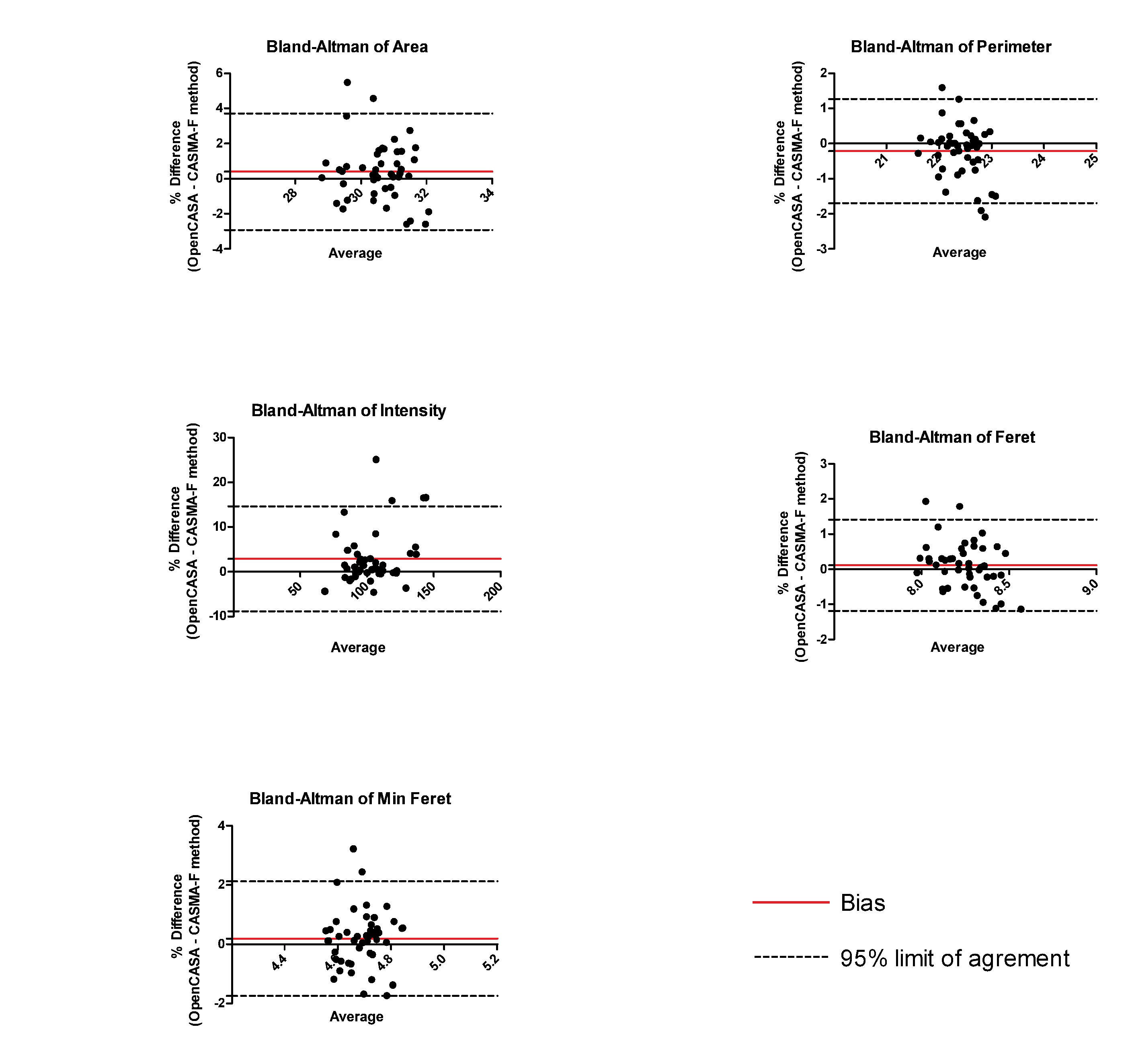

Supplement: S2 Fig — Results obtained for morphometry in the validation test. (TIF) [file pcbi.1006691.s002.tif]

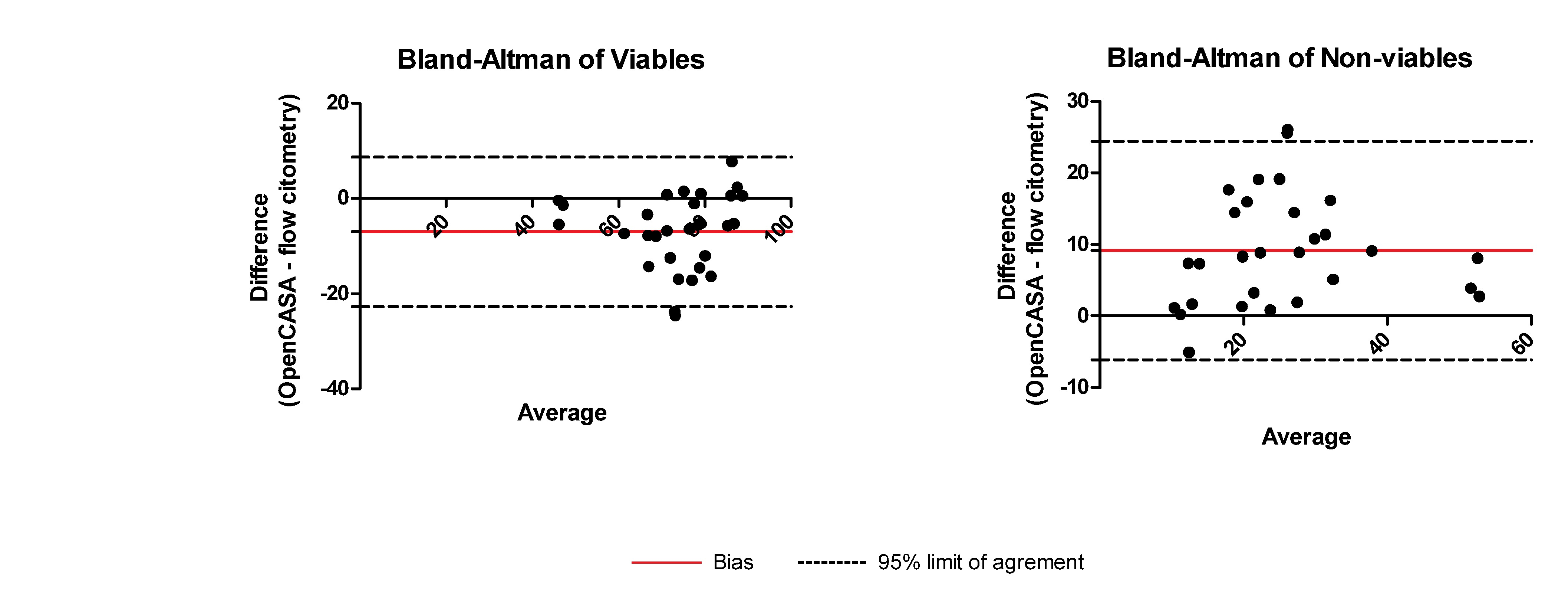

Supplement: S3 Fig — Results obtained for viability in the validation test. (TIF) [file pcbi.1006691.s003.tif]
