# Supplementary material for: OpenCASA: A new open-source and scalable tool for sperm quality analysis
Source: PLoS Comput Biol. 2019 Jan 18;15(1):e1006691. doi: 10.1371/journal.pcbi.1006691 (PMC6355034; doi:10.1371/journal.pcbi.1006691)

V1.0

Software Design Document

OpenCASA

Table of Contents

Introduction 1

Purpose 1

Scope 1

System Architecture 2

Architectural Design 2

Decomposition Description 2

Data Design 4

Component Design 6

Algorithms 6

Automatic threshold 6

Object detection 6

Track identification 7

Some useful activity diagrams 8

Chemotaxis Analysis 8

Chemotaxis Index Analysis 8

Chemotaxis Experiment Analysis 9

Chemotaxis Simulated Experiment Analysis 9

Motility File Analysis 10

Motility Folder Analysis 10

Motility Multiple Folders Analysis 10

Image analysis Window 10

Introduction

Purpose

The purpose of this document is to provide an overall view of the OpenCASA software design and its implementation.

Scope

OpenCASA is an integrative project oriented to cell multi-analysis. The initial version has been designed to include five modules: motility, chemotaxis, viability, morphometry and simulation, but also to facilitate the inclusion of new modules:

- **Motility module** takes a video file (or multiple files) where a cell population has been recorded, tracks every cell along the video, and calculates different kinematic parameters for each track.
- **Chemotaxis module**, as motility module, takes a video file (or multiple files) where a cell population has been recorded, and analyses the bias in the directionality of the cells movement.
- **Viability module** allows the user to count different populations of stained cells according to their color (red or green) in sets of multiple images.
- **Morphometry module** allows the user to calculate different parameters related to cell morphometry in sets of images.
- **Simulation module** generates a video file of simulated cell populations that follow a persistent random walk model.

System Architecture

Architectural Design

The architecture has been designed to maximize the reutilization of code by different modules and to group different pieces of code in packages depending on their purpose.

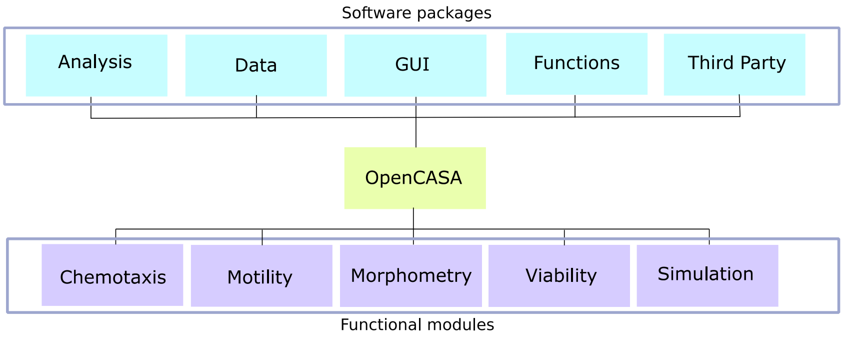

The initial design has been thought to have five packages:

Package	**Description**
Analysis	This package includes code directly related to particular analysis of each module.
Data	This package includes the declaration of all data structures designed.
GUI	This package includes the code related to the graphical user interface.
Functions	This package includes generic functions that could be used by every module, i.e. statistical functions, video processing or drawing functions.
Third Party	This package has been designed to include and locate third party code used in the project.

Decomposition Description

Here are explained in more detail all units included in each package (Data package will be explained in the following section):

Analysis Package	**Description**
Chemotaxis	This unit implements all functions related to chemotaxis analysis.
Motility	This unit implements all functions related to motility analysis.

Functions Package	Description
Computer Vision	This unit includes all functions related to image processing.
File Manager	This unit includes all functions used to manage files, directories, file names and file types.
Kinematics	This unit implements all methods used to analyse cell trajectories, like velocities or motility tests.
Paint	This unit includes all functions used to draw results on screen, like diagrams or trajectories over a video file.
Signal Processing	This unit includes a set of filters used to manipulate cell trajectories. For example, it includes a function to calculate the average path of a trajectory or to decimate when a high frame rate has been used to record the videos.
Trial Manager	This unit includes functions to extract trials from video files, simulate trials or to read/save trials from/to the file system.
Utils	This unit includes generic functions like dialogs or print tracks throw standard output.
Video Recognition	This unit includes all functions used to identify cell tracks over a video file.

GUI Package	Description
Chemotaxis Settings	This unit manages all the GUI elements used to show and set by the user all parameters involved in the chemotaxis analysis.
Image Analysis Window	This unit implements a generic window designed to image analysis. It includes various slide bars, buttons to navigate forward a backward, and a generic image area to show the current image.
Main Window	This unit implements the main window of the software. It includes a grid window with one button for each module available.
Morphometry Settings	This unit manages all the GUI elements used to show and set by the user all parameters involved in the morphometry analysis.
Morph Window	This unit inherits from Image Analysis Window and it is an adaptation to carry on morphometry analysis. Due to the type of analysis and the importance of the user interaction in this analysis, all functions for morphometry analysis are implemented here, and no specific file is included in Analysis package.
Motility Settings	This unit manages all the GUI elements used to show and set by the user all parameters involved in the motility analysis.
Settings Window	This unit integrates all settings from the different modules.
Viability Settings	This unit manages all the GUI elements used to show and set by the user all parameters involved in the viability analysis.
Viability Window	As Morph Window, this unit inherits from Image Analysis Window and it is an adaptation to carry on viability analysis. Due to the type of analysis and the importance of the user interaction in this analysis, all functions for viability analysis are implemented here, and no specific file is included in Analysis package.

Data Design

The data structures included in data package are:

Data structure	Description
Chemotaxis Params	This unit reads and writes to file system all parameters involved in the chemotaxis analysis. This parameters can be modified in Settings Window.
Morphometry Params	This unit reads and writes to file system all parameters involved in the morphometry analysis. This parameters can be modified in Settings Window.
Motility Params	This unit reads and writes to file system all parameters involved in the motility analysis. This parameters can be modified in Settings Window.
Viability Params	This unit reads and writes to file system all parameters involved in the viability analysis. This parameters can be modified in Settings Window.
Params	This unit is used internally by the software to set the parameters used in the execution of a specific module. When the user selects any module, this unit is set with the specific configuration for this module.
Serializable List	This unit is an extension of a standard ArrayList in which has been added the Serializable interface, in order to serialize and store data into file system.
Cell	This unit encapsulates all relevant information about a cell.
Trial	This unit stores all relevant information about a single file: tracks, identifier, file type, etc.
Simulation	This unit implements a generic interface that all simulations has to maintain.
Oscillatory Walker	This unit implements a particular simulation. The simulated cells oscillate following a sinusoidal movement.
Persistent Random Walker	This unit codifies a particular simulation. The simulated cells behave according to a persistent random walk model.

Other important structure that commonly appears in video analysis is the one used to store trajectories. When a video is analyzed, a list of tracks is returned. Each track is represented as a list of Data.Cell where each object holds the location (x, y) of the real cell at some specific frame.

The workflow of a video analysis is represented in the following diagram:

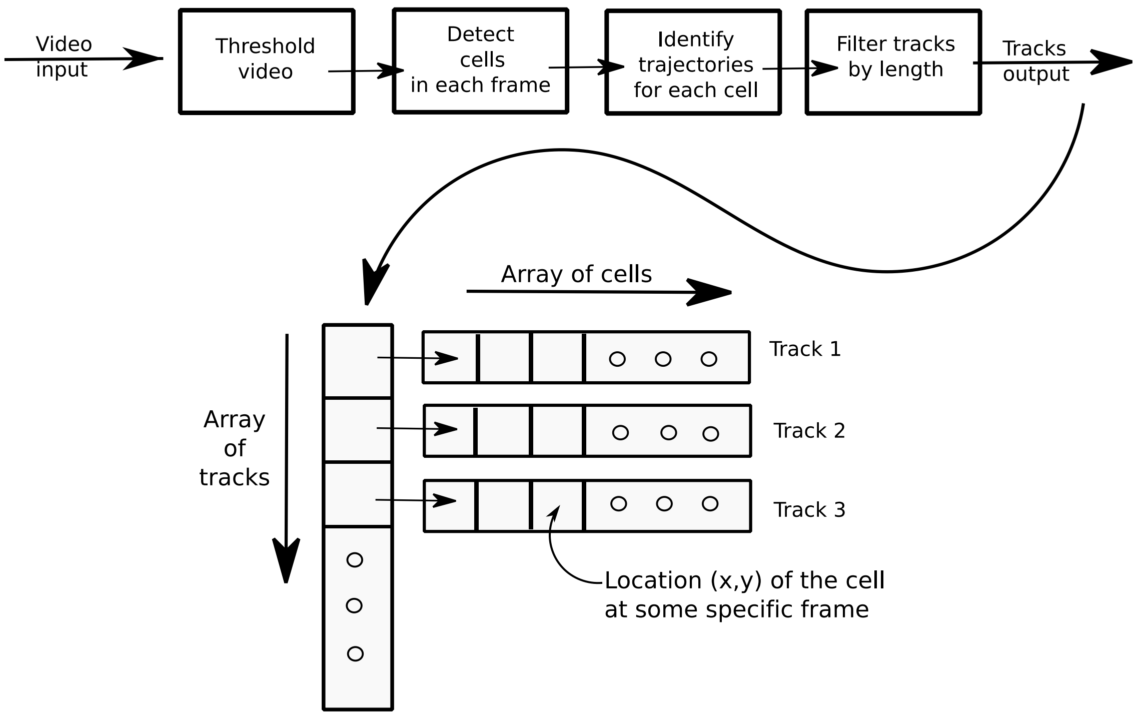

Component Design

Algorithms

Automatic threshold

In video analysis, once the program has converter the input video to grayscale, a threshold is applied to differentiate the objects of interest (i.e. cells) from background. In a video converted to grayscale, each pixel has a value between 0 and 255. Supposing that there are more pixels related to background than to cells, the method implemented takes the histogram of gray pixels and calculates the mean and standard deviation of the distribution. The threshold is set to 2 times the standard deviation.

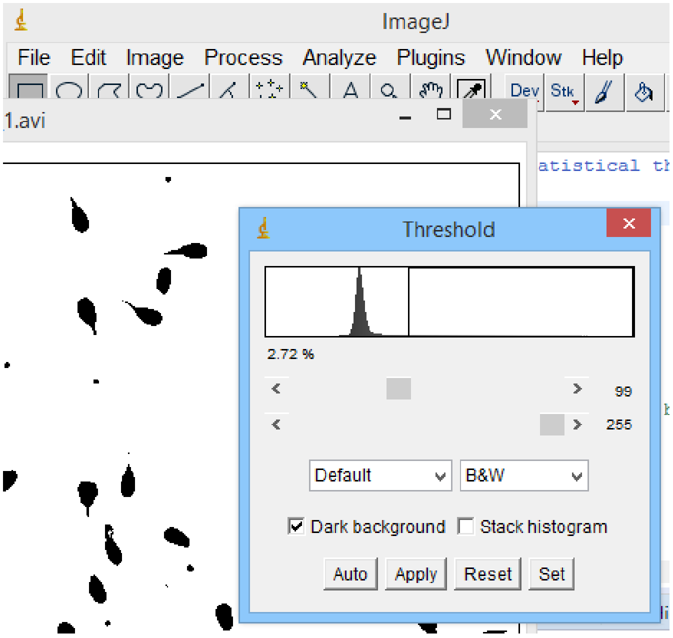

For image analysis, there is an option to calculate manually the threshold but also it is possible to apply the Otsu and Minimum automatic threshold methods.

Object detection

In order to detect objects in binary images it has been used the plugin ParticleAnalyzer included in ImageJ libraries.

More info at https://imagej.nih.gov/ij/developer/api/ij/plugin/filter/ParticleAnalyzer.html.

Track identification

The algorithms used to detect and identify tracks are the same ones implemented by Jonas Wilson-Leedy and Rolf Ingermann in the original CASA plugin for ImageJ.

Cell detection

Input: Video
Output: For each frame, it returns a list of all cells detected and their (x, y) location.

- Initialize Cells as an empty list.
- For each video frame:
    1. Create an empty list
    2. Identify all cells and their locations in the frame and add each cell to the list
    3. Put the list into the last Cells index
- Return Cells list

Track identification

Input: List of all cells for each frame (as list of lists)
Output: List of tracks

```
For each frame (F):
    Get Cell list identified in F
    Initialize the tracks' list as an empty list
    For each cell (C1) in F:
        If C1 is not already in a track:
            Initialize a track as an empty list
            Add C1 to the track
            currentCell = C1
            For each frame (Fn) in the following frames:
                For each cell (Cn) in Fn:
                    Calculate the distance between currentCell and Cn
                    If the distance is lower than the maximum displacement
                    allowed between frames and Cn is not already in a track:
                        If is not the first candidate:
                            Get the closest candidate to currentCell
                        end if
                    else if distance is lower than the maximum displacement
                    allowed between frames but Cn is already in a track:
                        Ignore
                    end if
                end for
                If there is a candidate:
                    Add this candidate to the track
                else:
                    Stop search
                end if
                currentCell=Cn
            end for
            Add the track to the tracks' list
        end if
    end for
end for
Return list of tracks
```

Some useful activity diagrams

Chemotaxis Analysis

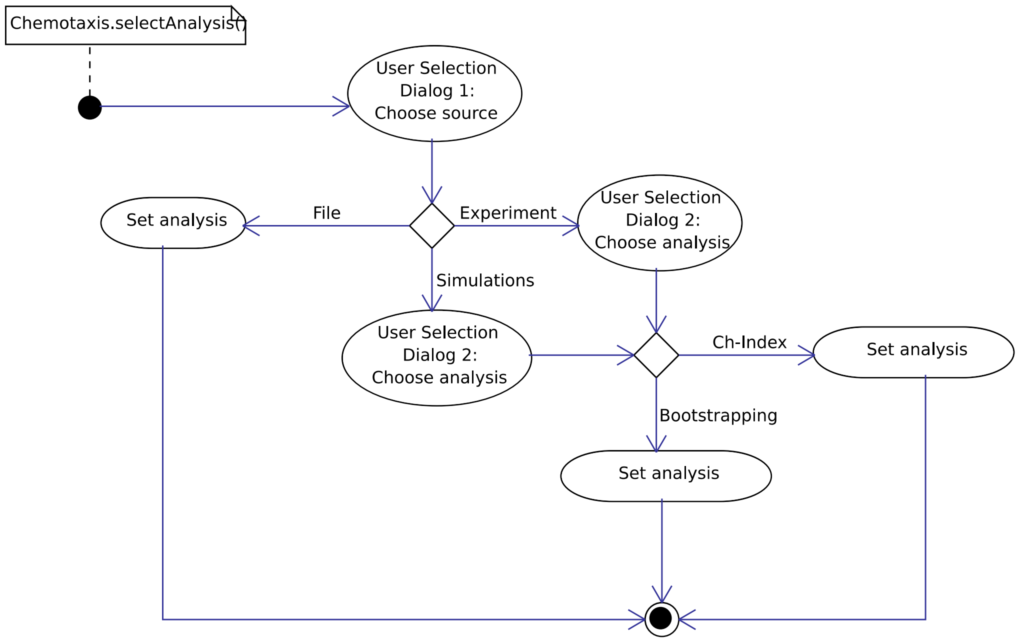

Chemotaxis Index Analysis

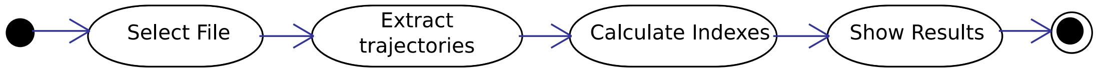

Chemotaxis Experiment Analysis

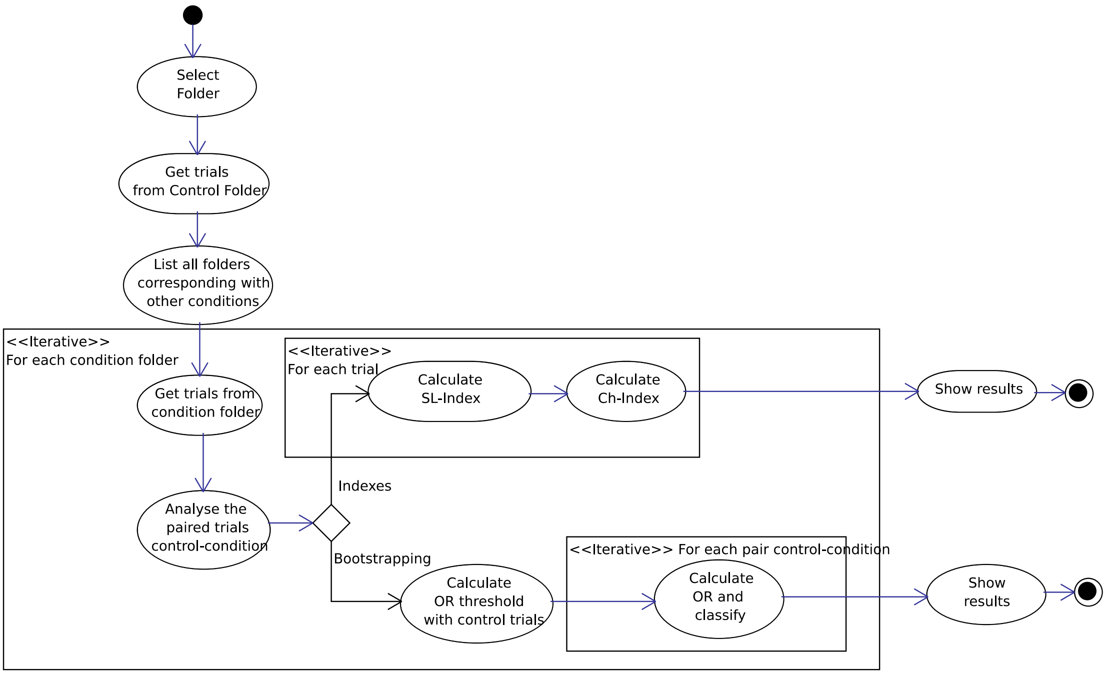

Chemotaxis Simulated Experiment Analysis

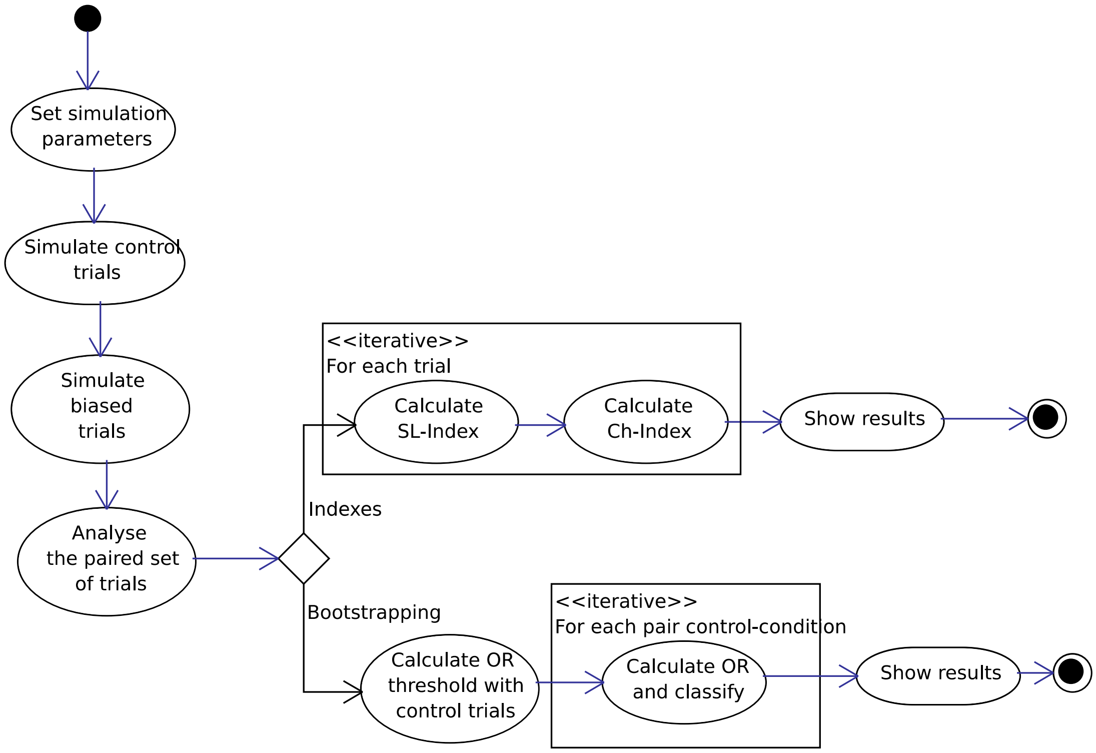

Motility File Analysis

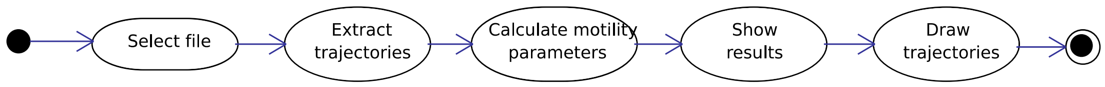

Motility Folder Analysis

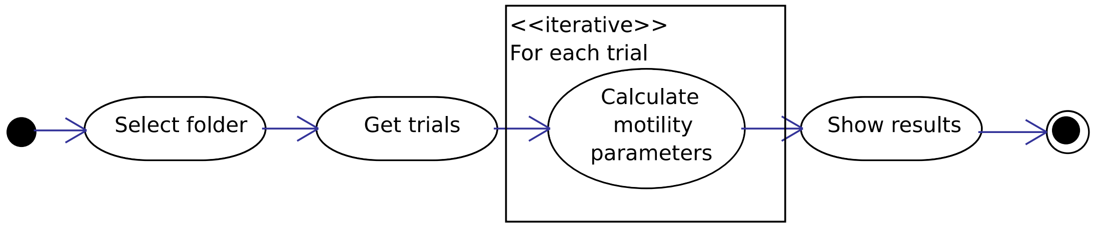

Motility Multiple Folders Analysis

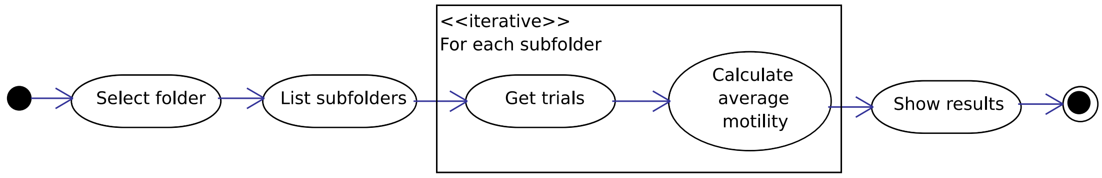

Image analysis Window

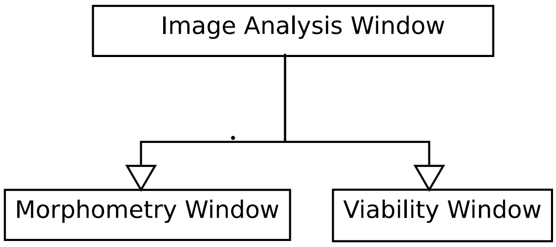

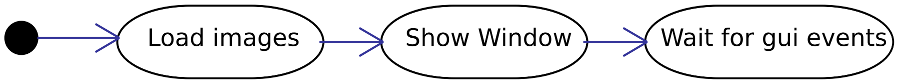

Supplement: S1 File — This document contains the specification of some algorithms, data structures and activity diagrams with the purpose to provide an overall view of the OpenCASA software design and its implementation. (PDF) [file pcbi.1006691.s004.pdf]
